# Potential Utility of Protein Targets of Cysteine-S-Nitrosylation in Identifying Clinical Disease Status in Human Chagas Disease

**DOI:** 10.3389/fmicb.2018.03320

**Published:** 2019-01-15

**Authors:** Maria Paola Zago, John E. Wiktorowicz, Heidi Spratt, Sue-Jie Koo, Natalia Barrientos, Aida Nuñez Burgos, Julio Nuñez Burgos, Facundo Iñiguez, Valentina Botelli, Ricardo Leon de la Fuente, Nisha Jain Garg

**Affiliations:** ^1^Instituto de Patología Experimental, CONICET-UNSa, Salta, Argentina; ^2^Department of Biochemistry and Molecular Biology, University of Texas Medical Branch (UTMB), Galveston, TX, United States; ^3^Institute for Human Infections and Immunity, University of Texas Medical Branch (UTMB), Galveston, TX, United States; ^4^Department of Preventive Medicine and Community Health, University of Texas Medical Branch (UTMB), Galveston, TX, United States; ^5^Department of Pathology, University of Texas Medical Branch (UTMB), Galveston, TX, United States; ^6^Servicio de Cardiología, Hospital San Bernardo, Salta, Argentina; ^7^Servicio de Cardiología, Programa de Medicina Interna, Hospital Papa Francisco, Salta, Argentina; ^8^Department of Microbiology and Immunology, University of Texas Medical Branch (UTMB), Galveston, TX, United States

**Keywords:** infectious disease, Chagas cardiomyopathy, *Trypanosoma cruzi*, S-nitrosylation, peripheral blood mononuclear cells, 2DE, mass spectrometry

## Abstract

*Trypanosoma cruzi (Tc)* infection causes Chagas disease (ChD) presented by dilated cardiomyopathy and heart failure. During infection, oxidative and nitrosative stresses are elicited by the immune cells for control the pathogen; however, excess nitric oxide and superoxide production can result in cysteine S-nitrosylation (SNO) of host proteins that affects cellular homeostasis and may contribute to disease development. To identify the proteins with changes in SNO modification levels as a hallmark of ChD, we obtained peripheral blood mononuclear cells (PBMC) from seronegative, normal healthy (NH, *n* = 30) subjects, and from seropositive clinically asymptomatic (ChD CA, *n* = 25) or clinically symptomatic (ChD CS, *n* = 28) ChD patients. All samples were treated (Asc+) or not-treated (Asc^−^) with ascorbate (reduces nitrosylated thiols), labeled with the thiol-labeling BODIPY FL-maleimide dye, resolved by two-dimensional electrophoresis (total 166 gels), and the protein spots that yielded significant differences in abundance or SNO level at *p*-value of ≤ 0.05_*t*−test/Welch/BH_ were identified by MALDI-TOF/TOF MS or OrbiTrap LC-MS/MS. Targeted analysis of a new cohort of PBMC samples (*n* = 10–14/group) was conducted to verify the differential abundance/SNO levels of two of the proteins in ChD (vs. NH) subjects. The multivariate adaptive regression splines (MARS) modeling, comparing differences in relative SNO level (Asc^−^/Asc+ ratio) of the protein spots between any two groups yielded SNO biomarkers that exhibited ≥90% prediction success in classifying ChD CA (582-KRT1 and 884-TPM3) and ChD CS (426-PNP, 582-KRT1, 486-ALB, 662-ACTB) patients from NH controls. Ingenuity Pathway Analysis (IPA) of the SNO proteome dataset normalized to changes in protein abundance suggested the proteins belonging to the signaling networks of cell death and the recruitment and migration of immune cells were most affected in ChD CA and ChD CS (vs. NH) subjects. We propose that SNO modification of the select panel of proteins identified in this study have the potential to identify ChD severity in seropositive individuals exposed to *Tc* infection.

## Introduction

Chagas disease (ChD), a neglected parasitic disease recognized as one of the top public health concern in the world, is endemic in Latin America and Mexico (Bonney, 2014). World Health Organization estimates suggest that ~6–7 million people worldwide are infected with *Trypanosoma cruzi* (*T. cruzi* or *Tc*) (World Health Organization, 2010). With an increase in migratory movements, additional millions of people are at risk of infection not only in Latin America, Mexico, and southern regions of the United States, but also in many countries of Europe where three million of migrants from American endemic areas are living (Bern et al., 2011; Tanowitz et al., 2016a; Monge-Maillo and Lopez-Velez, 2017). The presentation of high parasitemia and general febrile illness during acute phase can result in myocarditis related death in ~5% of the infected individuals. An asymptomatic phase occurs indeterminately, although ~30% of the infected people could develop chronic Chagas disease (ChD) that results in cardiomyopathy and heart failure (Machado et al., 2012; Bonney et al., 2019). Some patients also exhibit megacolon and digestive disorders that affect the quality of life. Currently, *T. cruzi* infection is treated with benznidazole or nifurtimox. These drugs are effective in children presenting the acute infection phase (Perez-Molina and Molina, 2018), but exhibit limited efficacy and high toxicity in infected adults that are at risk of developing heart failure (Viotti et al., 2014). The current methods of detecting infection is by microscopic examination of blood smears, serology, or PCR (Ribeiro et al., 2012), but no methods currently exist to track or predict ChD progression (Balouz et al., 2017).

*T. cruzi* activates the production of oxidants (e.g., superoxide, nitric oxide) from enzymatic and mitochondrial sources in immune and non-immune cells (Lopez et al., 2018). While the immune activation of reactive oxygen species (ROS) and nitric oxide are important for controlling *T. cruzi*, non-resolution of these responses can damage the host (Tanowitz et al., 2016b; Lopez et al., 2018). Indeed, we have reported the markers of chronic oxidative and nitrosative stress were increased in both, the heart and peripheral blood, tissues of the *T. cruzi*-infected rodents and humans during the development and progression of ChD (Wen et al., 2006; Wan et al., 2016). Nevertheless, the host proteins that are susceptible to oxidative/nitrosative stress-mediated changes in structure, function, or stability during ChD progression are not known.

The protein S-nitrosylation (SNO) is an ubiquitous, redox reversible, post-translational modification of cysteine residues that occurs in presence of excessive oxidative and nitrosative stress (Htet Hlaing and Clement, 2014). This modification is shown to influence the translocation, localization, and stability of proteins, and thus can drive the gain as well as loss of function in biological and disease conditions (Moldogazieva et al., 2018).

We previously reported that peripheral blood mononuclear cells (PBMC) carry differential protein abundance signatures of ChD (Garg et al., 2016) and idiopathic heart failure (Koo et al., 2016). Protein profiling of ChD patients' PBMC suggested that proteins related to immune cell migration and free radical synthesis and catabolism were differentially abundant by disease state (Garg et al., 2016). Further, a select number of differentially abundant proteins had the predictive value in distinguishing ChD patients vs. normal healthy contols. Such protein signatures of easily accessible cells may have potential to serve as biomarkers of ChD severity and the patients' response to treatment, to be verified in future studies.

In this study, we report the cysteine SNO fingerprint associated with Chagas disease development. For this, enrolled volunteers were evaluated by cardiologists and grouped according to the severity of their cardiac anomalies following the modified Kuschnir classification criteria based on physical exam, electrocardiography, and transthoracic echocardiography (Sanchez-Montalva et al., 2016). The PBMC samples of ChD patients and normal healthy volunteers were treated (or not treated) with ascorbate, and labeled with thiol-labeling BODIPY® FL *N*- (2-aminoethyl) maleimide (BD) dye. BD labels all proteins containing one or more cysteine residues, and it is capable of detecting >92% of the human proteins (Tyagarajan et al., 2003; Wiktorowicz et al., 2017). The advantage of this approach is the quantitative labeling of protein cysteine residues in a stable and specific fashion to analyze them through two-dimension gel electrophoresis (2D-GE) and mass spectrometry (Wiktorowicz et al., 2017). Our predictive modeling of the proteome datasets suggest that proteins belonging to the signaling networks of (a) cell death and (b) proliferation and recruitment/migration of immune and fibroblast cells were most affected by S-nitrosylation in ChD CA and ChD CS (vs. NH) subjects. We discuss the potential significance of differential SNO protein profile in identifying the severity of ChD in infected patients.

## Materials and Methods

### Study Population and Statement of Ethics

The institutional review board at the University of Texas Medical Branch at Galveston (IRB 04-257), the ethics committee of the School of Health Sciences at Universidad Nacional de Salta, and the institutional review board of Health Ministry of Salta, Argentina reviewed the study protocol, and all studies were carried out after approval of the human subjects study protocol by the three entities. A written informed consent was obtained from the individuals invited to participate before collection of blood samples. The study enrolled volunteers that came to seek medical assistance at the public hospitals in Salta city, Salta, Argentina. All samples were coded and de-identified before being provided for research purposes.

### Human Samples

Sera samples from all enrolled volunteers were analyzed for anti-*T. cruzi* antibodies by two tests. Chagatest-ELISA recombinante v.4.0 assay detects antibodies to six recombinant proteins expressed in a variety of *T. cruzi* isolates. Chagatest-HAI assay detects sera antibody based heamaglutination of red blood cells sensitized with cytoplasmic and membrane antigens of *T. cruzi*. Both assays were carried out following protocols provided by the manufacturer (Wiener, Rosario, Argentina). Samples qualified as positive by both tests were identified as seropositive, and these individulas were grouped as ChD subjects. Later, chest X-ray, electrocardiography (ECG, 12-lead at rest, and 3-lead with exercise) and transthoracic echocardiography were performed to assess the heart pathology and function to categorize enrolled individuals according to the Kuschnir classification (Sanchez-Montalva et al., 2016). Briefly, seropositive individuals with minor to no ECG abnormalities, no changes in ventricular walls, and normal ejection fraction (range: 55–70%) were grouped as ChD clinically asymptomatic (CA) subjects. Seropositive individuals with a degree of ECG abnormalities, cardiomegaly, systolic dysfunction (ejection fraction: < 55%), left ventricular dilatation (diastolic diameter ≥57 mm), and/or potential signs of heart failure were categorized as ChD clinically symptomatic (CS) patients. Seronegative subjects with no history or clinical symptoms of heart disease were enrolled as normal healthy (NH) controls (Garg et al., 2016).

### Purification of PBMC, Labeling With BODIPY, and Protein Resolution by Two-Dimensional Gel Electrophoresis (2D-GE)

We enrolled ChD CA (*n* = 25, 46% males, age: 49.8 ± 9 years) and ChD CS (*n* = 28, 53% males, age: 53 ± 10.6) patients and NH (*n* = 30, 50% males, age: 39 ± 16 years) subjects in the study. Blood samples (10–15 ml) were collected from all enrolled patients in the heparinized, BD Vacutainer CPT Cell Preparation Tubes. The tubes were centrifuged in a swing bucket rotor at 400 × g, 4°C for 10 min. After removing the upper plasma layer, packed blood cells were diluted with ice-cold phosphate buffered saline (PBS), layered on to The FICOLL Hypaque™ density gradient, and centrifuged at 4°C at 400 × g for 40 min. The PBMC layer at the plasma-Ficoll interface was transferred to a new tube, washed with ice-cold Hank's balanced salt solution, and stored at −80°C until used (Garg et al., 2016; Koo et al., 2016).

The PBMC pellets were lysed in urea buffer (50 mM Tris pH 7.5 containing 7 M urea, 2 M thiourea, and 2% CHAPS), and all PBMC lysates were analyzed by Lowry method to evaluate the protein concentrations, and by L8800 amino acid analyzer (Hitachi High Technologies, Pleasanton, CA) to determine the cysteine (cysteic acid) levels (Koo et al., 2016). The samples were briefly centrifuged to remove cellular debris, and then split into two aliquots. Aliquot A was treated with 6 mM ascorbate (Asc+) for 1 h to reduce SNO, and aliquot B was treated with 100-μM neocuproine for 1 h to preserve SNO (Asc^−^). Both aliquots of each sample were separately dialyzed in dialysis tubes (3.5 kDa MWCO) against urea buffer. The dialyzed samples were labeled for 2 h with BODIPY® FL *N*- (2-aminoethyl) maleimide (BD, Life Technologies, Grand Island, NY) that was added at saturating concentration (BD: cysteine concentration, 60: 1 ratio). All incubations were carried out at room temperature while protecting the samples from light, and the reaction was stopped with ß-mercaptoethanol added in 10: 1 molar excess to the BD dye (Pretzer and Wiktorowicz, 2008; Wiktorowicz et al., 2011, 2017).

The BD-labeled, Asc+ and Asc^−^ PBMC lysates (100-μg each) were separated by 2D-GE, employing an IPGphor multiple sample isoelectric focusing (IEF) device (GE Healthcare, Chicago, IL) in 1st dimension, and the Criterion Dodeca cell (Bio-Rad, Hercules, CA) in 2nd dimension, following the protocol previously described by us (Garg et al., 2016).

### Gel Fixing, BD Imaging, and Image Processing

All gels were fixed in 20% methanol/7% acetic acid/10% acetonitrile for 1 h, washed with 20% ethanol / 10% acetonitrile overnight, washed with dH_2_O, and BD-labeled proteins were imaged at 100 μm resolution (Ex_488nm_/Em_520nm_) by using the Typhoon Trio Variable Mode Imager (GE Healthcare). The voltage was set to result in 85–99% of the saturation level for the most abundant protein on the gel. Gel images taken under the BD-specific filters were used to obtain the spot-specific data (Wiktorowicz et al., 2017).

A reference gel from the set of Asc+ gels was selected by Totallabs Ltd SameSpots software (Newcastle, UK). The reference gel was stained overnight with Sypro Ruby Stain (Life Technologies), and imaged at Ex_488nm_/Em_560LPnm._ The reference gel image (100 μM resolution) was used to ensure the detection of all protein spots irrespective of presence or absence of cysteine residues and to define the spot boundaries. The matching spot volumes from BD-stained Asc+ and Asc^−^ gels were used to obtain the quantitative spot data (Wiktorowicz et al., 2017).

### Quantification of SNO Levels

In total, 166 BODIPY-stained 2DE gels [two gels with either Asc^+^ or Asc^−^ protein lysates for each sample from the NH (*n* = 30), ChD CA (*n* = 25), and ChD CS (*n* = 28) subjects] were scanned and anayzed using the Totallabs Progenesis SameSpots™ software. The spot volumes were subjected to statistical analysis by using built-in tools of Totallab SameSpots software. The data were found to be normally distributed, and the differential protein abundance and/or SNO levels for all protein spots between any two groups was subjected to statistical analysis by Student's *t*-tests with Welch's correction for unequal variances. Also, to account for the false discovery rate, Benjamini–Hochberg (B-H) multiple hypotheses testing correction was applied and significance was accepted at *p*-value ≤ 0.05 (Koo et al., 2016; Wiktorowicz et al., 2017).

The SNO modification levels per protein spot from each cohort (NH, ChD CA, ChD CS) were quantified by calculation of the ratio of fluorescence units from Asc^−^ and Asc^+^ signal for each spot (SNO signal/total protein signal = Asc^−^/Asc^+^) (Wiktorowicz et al., 2011). Because SNO modification prevents Cys from being labeled by the BD fluor; a Asc^−^/Asc^+^ ratio of < 1 or a negative value (after log transformation) indirectly reflects the “percentage” of SNO modified protein in the spot.

The final calculation of the SNO modification per unit of protein was carried out according to the following formula, called Ratio of ratios (RoR):

$$\text{Ratio of ratios}=\frac{\left[{\text{BD}}_{\text{Asc}-}^{\text{Exp}}{\text{/BD}}_{\text{Asc}-}^{\text{Ctrl}}\right]}{\left[{\text{BD}}_{\text{Asc}+}^{\text{Exp}}{\text{/BD}}_{\text{Asc}+}^{\text{Ctrl}}\right]}=\frac{\Delta [\text{Cys}-\text{NO}]}{\Delta [\text{protein}]}$$

By adjusting for abundance changes, the RoR is used to establish true SNO-specific changes in experimental sample with respect to controls. Moreover, it is important to note that since SNO modification prevents the Cys-BODIPY labeling, a negative value indicates an increase in SNO level (and vice versa) in the sample (Wiktorowicz et al., 2017).

### Matrix Assisted Laser Desorption Ionization-Time of Flight (MALDI-TOF)/Mass Spectrometry (MS) for Protein Identification

The protein spots that were identified to be differentially abundant and/or differentially SNO-modified (*p* < 0.05) between any two groups were subjected for mass spectrometry identification as we have previously described (Dhiman et al., 2012; Wen et al., 2012). Briefly, protein spots (1 mm) on the 2D gels were picked robotically (ProPick II, Digilab, Ann Arbor, MI). Proteins were in-gel digested with 1% trypsin/25 mM NH_4_HCO_3_ at 37°C for 6 h. Peptide mixtures (1 μl) were directly spotted onto the MALDI TOF MS/MS target plate with 1 μl of alpha-cyano-4-hydroxycinnamic acid matrix solution (5 mg/ml in 50% acetonitrile) and analyzed by using an AB Sciex, TOF/TOF 5800 Proteomic Analyzer (Foster City, CA). The MS and MS/MS spectral data were acquired and analyzed by Applied Biosystems software package that included 4000 Series Explorer (v.3.6 RC1) with Oracle Database Schema (v.3.19.0) and Data Version (3.80.0) (Foster City, CA). The instrument was operated in a positive ion reflectron mode (focus mass: 1,700 Da, mass range: 850–3,000 Da), and 1,000–2,000 laser shots were acquired and averaged from each protein spot. A peptide mixture with the reference masses 904.468, 1296.685, 1570.677, and 2465.199 was utilized for automatic external calibration. Following MALDI MS, MALDI MS/MS was performed on 5–10 abundant ions from each protein spot. A 1 kV positive ion MS/MS method was used to acquire data under post-source decay (PSD) conditions. The instrument precursor selection window was ±3 Da. Automatic external calibration was performed by using reference fragment masses 175.120, 480.257, 684.347, 1056.475, and 1441.635 (from precursor mass 1570.700) (Dhiman et al., 2012; Wen et al., 2012).

The MS and MS/MS spectral data were searched against the UniProt human protein database (35,208,664 residues; 87,656 sequences; last accessed: July 12, 2016) by using AB Sciex GPS Explorer (v.3.6) software in conjunction with MASCOT (v.2.2.07, Matrix Science, London, UK) (Garg et al., 2016; Koo et al., 2016). The parameters used for MS peak filtering included a mass range of 800–3,000 Da, minimum S/N filter = 10, mass exclusion list tolerance = 0.5 Da, and exclusion of trypsin and keratin-containing compounds with masses of 842.51, 870.45, 1045.56, 1179.60, 1277.71, 1475.79, and 2211.1 daltons. The parameters for MS/MS peak filtering included a minimum S/N filter = 10, maximum missed cleavages = 1, fixed modification of carbamidomethyl (C), variable modifications due to oxidation (M), precursor tolerance = 0.2 Da, MS/MS fragment tolerance = 0.3 Da, mass = monoisotopic, and peptide charges = +1. The significance of a protein match, based on the MS and the MS/MS data from several precursor ions, was presented as a Protein Score with a confidence cutoff of ≥ 62. Some of the protein spots (|Fold change|≥ 1.5) identified with low confidence by MS/MS were subjected to LTQ OrbiTrap Fusion LC-MS analysis (Thermo Fisher Scientific, Waltham, MA) for protein identification (Garg et al., 2016; Koo et al., 2016).

### Enzyme-Linked Immunosorbent Assay (ELISA) and Biotin Switch Assay

A new set of PBMC samples from NH, ChD CA, and ChD CS subjects (*n* = 10–14 per group) were lysed by agitation for 30 min at 4°C in S-nitrosylation block buffer (Cayman Chemicals, Ann Arbor MI), and protein concentrations were determined by the Bradford method (Bio-Rad). The samples were divided into two aliquots; aliquot A was used to quantify the abundance of the target protein and aliquot B was processed to measure its SNO level.

Sandwich ELISA kits were used to quantify the changes in abundance of actin gamma (Reddot Biotech, Kelowna, Canada; detection range: 0.015–10 ng/mL) and filamin A (LifeSpan Biosciences, Seattle WA; detection range: 1.88–120 ng/mL) polypeptides by following manufacturer's instructions. In brief, aliquot A of PBMC lysates (2 μg in 100 μL/well) were loaded onto 96-well plates pre-coated with target-specific antibody. After 2 h incubation at 4°C, plates were aspirated and sequentially incubated with biotin-conjugated anti-target 2nd antibody (1: 100 dilution), avidin-conjugated horseradish peroxidase (HRP) (1: 100 dilution). The plates were washed between each reagent addition, color was developed with TMB substrate, and the change in absorbance was recorded at 450 nm by using a spectrophotometer (Spectramax 190, Molecular Devices, Sunnyvale, CA). A standard curve was prepared by using recombinant proteins and target protein concentration was plotted as pg per μg total protein (Koo et al., 2016).

The SNO modification levels of actin gamma and filamin A were assessed in aliquot B of each sample by the biotin switch assay. Briefly, free SH (thiol) groups in each sample were blocked with methyl methanethiosulfonate (MMTS), and then protein SNO bonds present in the sample were converted to thiols via transnitrosation with ascorbate. The newly formed SH groups were then labeled by S-biotinylation with biotin-HPDP by using a biotin switch-based, S-Nitrosylated Protein Detection Assay Kit (Cayman Chemicals) following the instructions provided by the manufacturer. Then, 96-well plates pre-coated with antibodies against actin gamma or filamin A were incubated for 2 h with biotin-derivatized protein lysates (2 μg in 100 μL/well). Plates were washed to remove the unbound proteins and then incubated for 10 min at room temperature with the avidin-conjugated HRP (1:3,000 dilution, BioLegend, San Diego, CA). The TMB substrate was added, and the change in absorbance reflecting the levels of biotin-bound SNO-modified actin gamma or filamin A was measured by spectrophotometry (Koo et al., 2016).

### Multivariate Adaptive Regression Splines (MARS) Analysis of the Proteome Datasets

MARS is a non-parametric regression procedure that automatically models non-linearities and interactions between variables (Friedman and Roosen, 1995; Austin, 2007). The input data were the Asc^−^/Asc+ (i.e., DSNO) values for the protein spots derived from each of the Asc^−^ and ASC+ gels of the NH (*n* = 30), ChD CA (*n* = 25), and ChD CS (*n* = 28) PBMC lysates. By using the R (R Foundation, Vienna, Austria) and SPSS (v.20, IBM Corporation, Armonk, NY) software, datasets were log2 transformed, screened by Student's *t*-test with Welch's correction (for non-equal variances amongst the groups), and Benjamini–Hochberg multiple hypothesis testing corrections were ran for the adjusted *p*-values. The significantly different protein spots between any two groups with *p*-value of ≤ 0.05_*t*−test/Welch/BH_ were used as input for MARS to model the predictive value of the selected variables in identifying ChD (vs.NH) patients. To avoid overfitting of the data, we employed two approaches: (1) 10-fold cross-validation (CV), allowing the same number of maximum basis functions as were the differentially SNO-modified spots at *p*-value of ≤ 0.05 (with 1 max interaction term) (Liu et al., 2017); and (2) training/testing approach in which randomly selected proteome datasets from 80% of the individuals in each group were utilized to create the model and the datasets from the remaining 20% of the subjects in each group were used to assess the fit of the model (Dobbin and Simon, 2011). The receiver operator characteristics (ROC) curves were developed to examine the sensitivity and specificity of the identified models.

### Ingenuity Pathway Analysis (IPA)

To assess the biological meaning of the proteome datasets, we used the IPA web-based application (Ingenuity Systems, Redwood city, CA) (Thomas and Bonchev, 2010). Briefly, the RoR values for SNO-modified proteins in ChD and control PBMCs were uploaded in the IPA to retrieve biological information, such as gene name, subcellular location, tissue specificity, function, and association with disease, etc. from the literature. Then the datasets were integrated to define networks and signaling pathways allowing us to understand the significance of data or candidate biomarkers in the context of a larger biological system (Garg et al., 2016; Koo et al., 2016).

## Results

### 2DE/MS Identification of Changes in PBMC SNO Proteome in Human Chagas Disease

**Figure 1:**
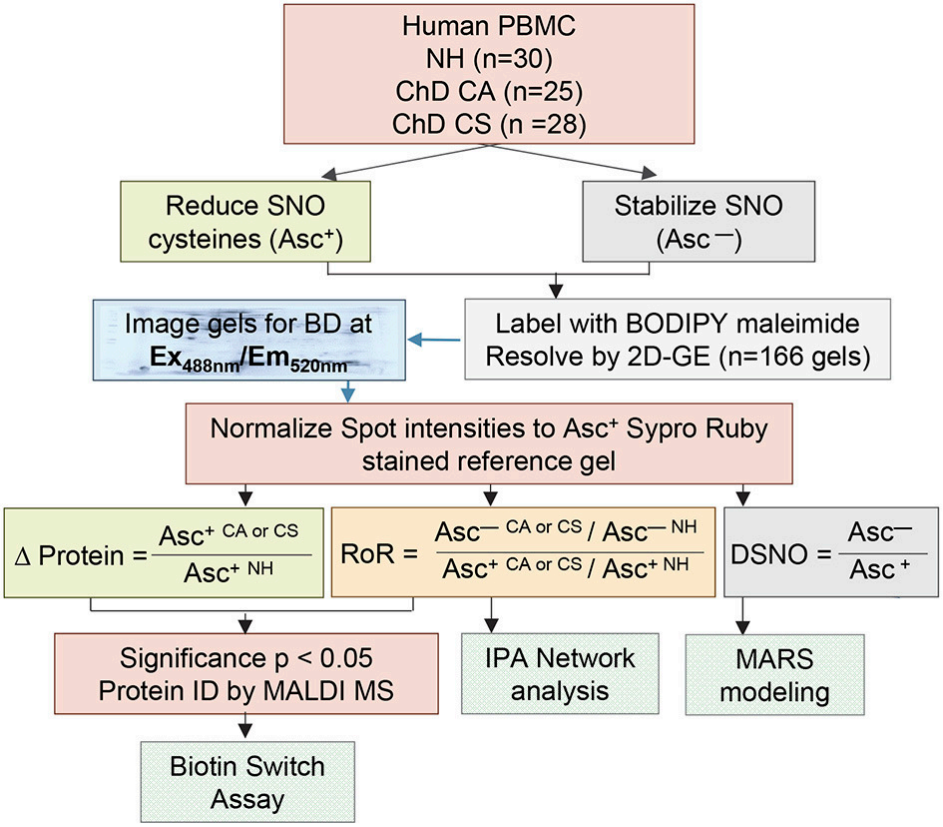
Schematic of work flow. Human PBMCs were obtained from volunteers categorized as seropositive, Chagas disease (ChD), clinically asymptomatic (ChD CA, *n* = 25) patients, seropositive, ChD clinically symptomatic (ChD CS, *n* = 28) patients, and seronegative, normal healthy (NH, *n* = 30) controls. Each sample was divided into two aliquots, and SNO cysteines were reduced with ascorbate (Asc+) in one aliquot, and stabilized with neocuproine in the 2nd aliquot (Asc ^−^). All sample aliquots were labeled with BODIPY FL *N*- (2-aminoethyl) maleimide (labels reduced cysteine) and resolved by 2-dimensional gel electrophoresis. Gel images were obtained with BD specific filter. A reference gel stained with Sypro Ruby was used to mark the spot boundaries, and the spot boundaries on all the BD labeled gels were normalized to Sypro Ruby stained reference gel. Ratiometric calculation of differential protein abundance from BODIPY-fluorescence units in Asc+ aliquots (CA or CS vs. NH) was performed for all protein spots. The relative percentage of each protein spot that was S-NO modified was quantitated by calculation of the ratio of fluorescence units from Asc–/Asc+ aliquots for each experimental condition (DSNO = Asc^−^/Asc+). The ratio of ratios (RoR) was calculated to obtain the change in S-NO levels in ChD patients (vs. NH controls) after normalizing for the changes in protein abundance. Protein spots that changed in abundance or S-NO modification at *p* ≤ 0.05 were submitted to mass spectrometry analysis for protein identification. Log2 transformed DSNO datasets were used for MARS modeling and RoR datasets were analyzed by Ingeunity pathway analysis (IPA). Selected proteins were confirmed for differential abundance and S-NO modification levels by ELISA/ Biotin switch assay.

**Figure 2:**
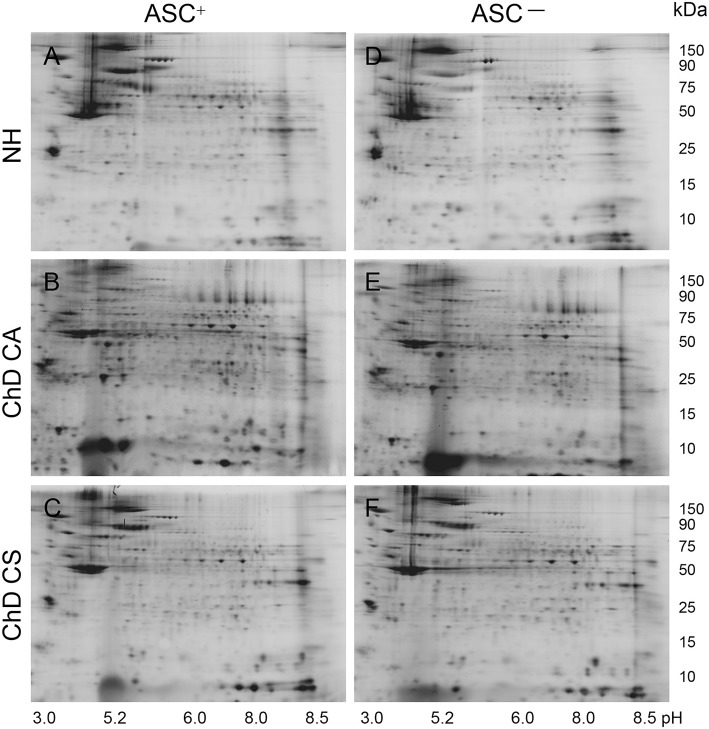
Representative two-dimensional gel images of protein spots in PBMC of Chagas disease subjects. BD-labeled PBMC lysates were separated in 1st dimension by isoelectric focusing on 11 cm non-linear pH 3–11 immobilized pH gradient strips, and in 2nd dimension by sodium dodecyl sulfate polyacrylamide gel electrophoresis (SDS-PAGE) on an 8–16% gradient gel. Gel images were obtained at 100 μm resolution to quantify BD-labeled proteins (Ex_488nm_/Em_520 ± 15nm_). Shown are the representative gel images of Asc+ **(A–C)** and Asc^−^ **(D–F)** PBMC lysates from NH **(A,D)** controls, and ChD CA **(B,E)**, and ChD CS **(C,F)** patients. Approximate size of the proteins in kDa (vertical) and pI ranges (horizontal) are noted.

A scheme of the steps involved in SNO proteomic analysis is presented in Figure 1. Each PBMC lysate was divided into two aliquots; aliquot A being reduced with ascorbate (Asc+, makes all cysteine residues available for BD labeling) and aliquot B (Asc^−^) was treated with neocuproine to preserve the SNO-modified cysteines. Labeling of the PBMC lysates with BD at saturating concentrations resulted in a no non-specific labeling. Further, BD had no effect on the isoelectric point and mobilities of the proteins/peptides, and it offered a highly sensitive method (detection limit: 5 fmol) to detect and quantitate the protein spots over a linear dynamic range of four orders of magnitude. The BD-labeled Asc^+^ and Asc^−^ PBMC lysates of NH controls (*n* = 30), and of ChD, clinically asymptomatic (CA, *n* = 25) and ChD, clinically symptomatic (CS, *n* = 28) patients were resolved on a total of 166 2D gels. Representative Asc^+^ and Asc^−^ gel images from each group are shown in Figures 2A–F. A reference gel stained with Sypro Ruby detected 635 protein spots within the relative molecular sizes of 10 to 250 kDa. The protein spot intensities on BD-labeled Asc+ and Asc^−^ NH, ChD CA, and ChD CS gels were normalized against the reference gel, and the data were analyzed in pair-wise manner. The protein spots that were changed in abundance and/or in SNO modification levels at *p* ≤ 0.05_*t*−test/Welch/BH_ between any of the two groups were submitted to MALDI-TOF/TOF MS analysis for protein identification.

**Figure 3:**
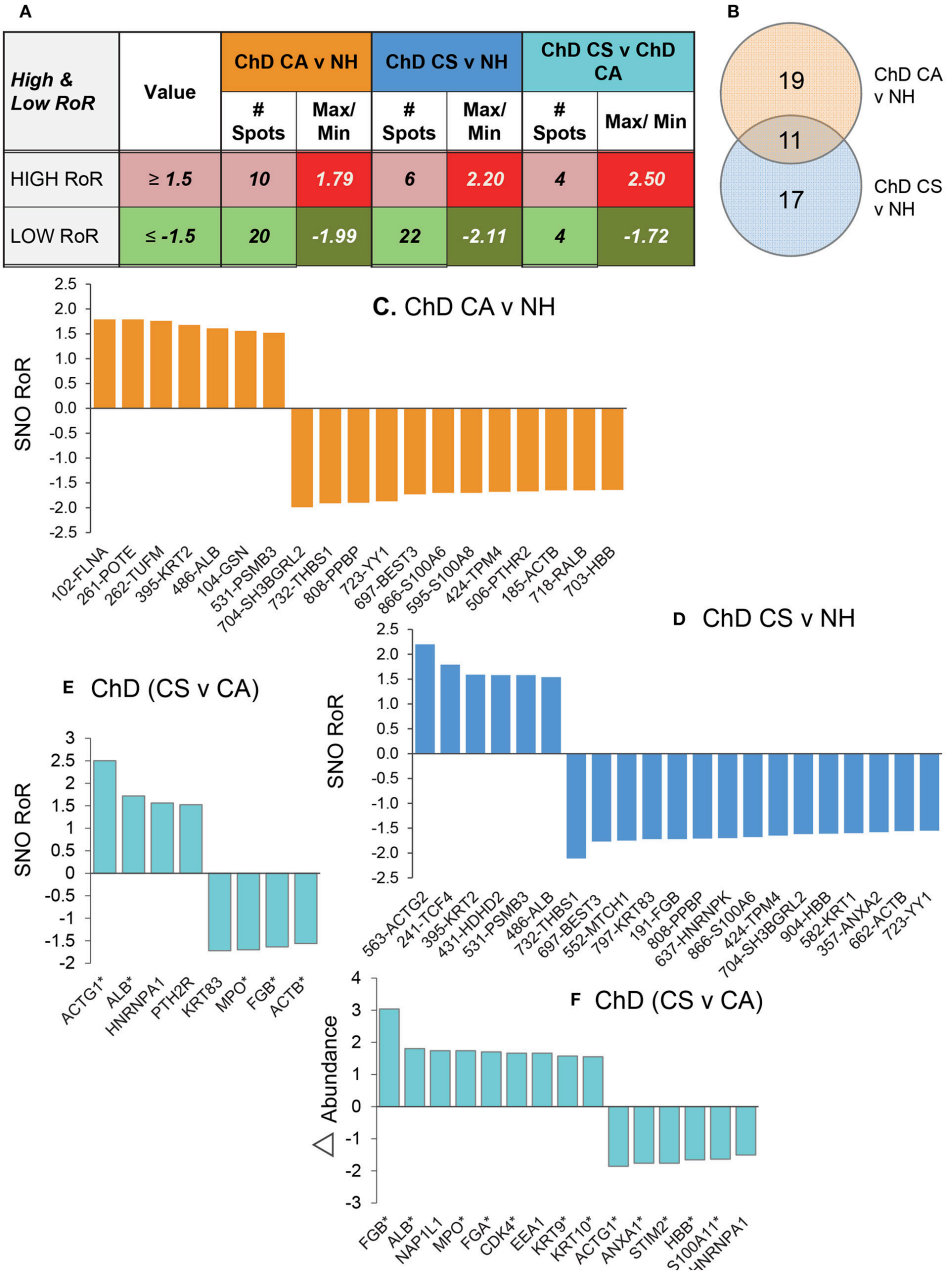
PBMC SNO signature in Chagas disease. **(A)** Number of protein spots that were changed in SNO levels (after normalizing for change in abundance) in ChD CA and ChD CS subjects with respect to NH controls (*p* ≤ 0.05) were calculated by ratio of ratio (RoR) method. Different intensity of red and green colors are in correspondence with Table 1. The darker shade means the higher values. **(B)** Venn diagram. Shown are the number of protein spots that were uniquely SNO modified (calculated by RoR method) in Chagas disease clinically asymptomatic and Chagas diease clinically symptomatic subjects. **(C–E)** Bar graphs show the RoR values of the top molecules in ChD CA vs. NH **(C)**, ChD CS vs. NH **(D)** and ChD CS vs. ChD CA **(E)** patients. A negative RoR indicates increased SNO modification and a positive RoR indicates increased reduction of protein thiols. The * next to protein name indicates multiple protein spots. **(F)** Proteins spots that also changed in abundance between ChD CS vs. ChD CA subjects (≥1.5-fold) are shown.

Of the 312 protein spots submitted to mass spectrometry, 249 protein/peptide spots were correctly identified (Table 1). Of these 12, 6 and 8 protein spots exhibited a change in Asc^−^/Asc+ ratio (i.e., DSNO value) that indirectly reflects the “percentage” of SNO modified protein in the spot (fold change: ≥1.5 at *p*-value of ≤ 0.05_*t*−test/Welch/BH_) in the NH, ChD CA, and ChD CS subjects (Table 1). The RoR values for all protein spots were calculated to normalize the changes in SNO levels with respect to changes in abundance. The RoR values indicated that 30 (20 increased, –ve RoR/10 decreased, +ve RoR) and 28 (22 increased, –ve RoR/6 decreased, +ve RoR) protein spots were differentially SNO-modified (normalized to change in abundance) in ChD CA and ChD CS subjects, respectively, in comparison to NH controls (fold change: |≥1.5|, *p* ≤ 0.05_*t*−test/Welch/BH_, Figure 3A). The changes in Cys-SNO modification frequency (RoR values) of the protein spots ranged from 1.79- to −1.99-fold and 2.20- to −2.11-fold in ChD CA and ChD CS patients (vs. NH subjects), respectively (Figure 3A). Venn diagram showed that 11 protein spots changed in RoR values in both ChD CA and ChD CS patients (vs. NH controls), and 19 and 17 protein spots exhibited changes in RoR values uniquely associated with the ChD CA and ChD CS patients, respectively (Figure 3B). The top molecules that were significantly changed in RoR values in ChD CA and ChD CS subjects (vs. NH controls) are shown in Figures 3C,D. The top molecules that distinguished the RoR and abundance profiles of ChD CS patients with respect to ChD CA patients are presented in Figures 3E,F. Note that proteomic profile identified some of the protein spots (e.g., PSMB3, SH3BGRL2, ACTB, THBS1) that were significantly changed in RoR values in both ChD CA and ChD CS patient groups; and other proteins (e.g., ACTG, ALB, HNRNPA1, PTH2R, KRT83, MPO, FGB, and ACTB) exhibited significant differences in RoR values between ChD CA and ChD CS subjects (Figure 3E). Importantly, proteomic profiling identified FLNA and ACTG were uniquely changed in RoR values in ChD CA and ChD CS subjects, respectively (Figures 3C,D), and SNO ACTG (spot# 563) was also differentially expressed between CA and CS patients (Figure 3E), and appears to be a hallmark of ChD severity.

**Table 1:** Detailed S-Nitrosylation proteome profile of PBMC proteins in human Chagas disease^a^.

**2D-GE data**			**MALDI MS data^b^**			**Asc–/Asc+** **(DSNO)^c^**			**RoR (Ratio of Ratios)^d^**		
**Spot no**.	**pI**	**MW (kD)**	**Protein name**	**Accession no**.	**MS ID protein score**	**NH**	**CA**	**CS**	**CA vs. NH**	**CS vs. NH**	**CS vs. CA**
52	6.4	108	Isoform 1 of Vinculin = VCL	P18206	709	1.14	−1.11	−1.05	−1.27	−1.19	1.06
54	6.6	108	Isoform 1 of Vinculin = VCL	P18206-2	1,180	1.08	−1.27	1.15	−1.37	1.06	1.46
57	6.7	107	Isoform 1 of Vinculin = VCL	P18206-2	1,130	−1.03	−1.10	1.15	−1.07	1.19	1.26
58	6.8	107	Isoform 1 of Vinculin = VCL	P18206-22	1,210	−1.01	−1.05	1.03	−1.05	1.04	1.09
59	6.9	107	Vinculin = VCL	P18206	1,170	1.01	1.03	1.06	1.02	1.05	1.03
60	7.1	105	PAK4-inhibitor INKA2 = FAM212B	Q9NTI7	29	1.04	1.17	1.20	1.13	1.16	1.03
63	7.5	99	Vinculin = VCL	P18206	236	1.12	1.18	−1.10	1.06	−1.23	−1.30
69	5.4	99	Actin, cytoplasmic 1 = ACTB	P60709	347	−1.36	−1.09	−1.11	1.24	1.23	−1.01
79	6.5	96	Serum albumin = ALB	P02768	190	−1.28	1.09	1.03	1.39	1.32	−1.05
81	6.6	94	Keratin, type I cytoskeletal 9 = KRT9	P35527	188	−1.18	1.10	−1.02	1.29	1.15	−1.12
89	5.6	88	Isoform 1 of Vinculin = VCL	P18206-2	323	1.08	−1.11	1.09	−1.19	1.02	1.21
90	5.7	87	POTE ankyrin domain family member E = POTEE	Q6S8J3	135	−1.04	−1.01	1.03	1.03	1.07	1.05
91	5.8	87	Filamin-A = FLNA	Q5HY54	330	−1.13	1.02	−1.09	1.15	1.04	−1.11
92	6.9	87	Filamin-A = FLNA	Q5HY54	384	−1.17	1.18	−1.09	1.38	1.07	−1.29
93	5.9	87	Filamin-A = FLNA	Q5HY54	389	−1.21	1.06	−1.04	1.27	1.16	−1.10
94	6.0	87	Filamin-A = FLNA	Q5HY54	238	−1.24	1.06	−1.04	1.32	1.20	−1.11
95	6.8	87	Filamin-A = FLNA	Q5HY54	399	−1.19	1.16	−1.22	1.38	−1.02	−1.42
96	6.1	86	Filamin-A = FLNA	P21333	169	−1.21	−1.12	−1.01	1.08	1.20	1.11
97	6.2	85	Filamin-A = FLNA	Q5HY54	164	−1.26	−1.28	−1.18	−1.02	1.06	1.08
99	4.5	84	78 kDa glucose-regulated protein GRP78 = HSPA5	P11021	849	1.01	1.03	1.05	1.02	1.04	1.01
100	6.3	83	Filamin-A = FLNA	Q5HY54	325	−1.28	−1.24	−1.12	1.03	1.14	1.11
101	5.4	83	Actin, cytoplasmic 1 = ACTB	P60709	297	−1.36	−1.15	−1.07	1.18	1.28	1.08
102	6.7	83	Filamin-A = FLNA	Q5HY54	210	−1.26	1.41	−1.06	1.79	1.19	−1.50
103	6.4	82	Filamin-A = FLNA	P21333	238	−1.28	1.15	−1.08	1.47	1.18	−1.24
104	6.5	82	Isoform 2 of Gelsolin = GSN	P06396-2	182	−1.32	1.18	1.04	1.56	1.37	−1.14
118	5.6	69	Actin, cytoplasmic 2 = ACTG1	I3L3I4	269	−1.04	−1.15	−1.10	−1.11	−1.06	1.05
121	5.4	68	Actin alpha 1, skeletal = ACTA1	Q5T8M7	227	−1.32	−1.04	−1.11	1.27	1.18	−1.07
124	5.2	68	Isoform 2 of Heat shock cognate 71 kDa protein = HSPA8	P11142-2	325	1.04	−1.20	−1.13	−1.25	−1.18	1.06
127	9.1	68	Isoform 2 of Fibrinogen alpha = FGA	P02671-2	62	1.07	1.07	−1.22	1.00	−1.30	−1.31
131	5.4	68	Keratin, type I cytoskeletal 10 = KRT10	P13645	296	−1.15	1.01	−1.04	1.16	1.11	−1.05
136	8.8	68	Transketolase = TKT	B4E022	189	−1.03	−1.15	−1.20	−1.11	−1.17	−1.05
141	6.0	63	Serum albumin = ALB	H0YA55	167	−1.14	−1.52	1.13	−1.33	1.29	1.72
142	4.5	63	Tropomyosin 3 = TPM3	Q5VU59	743	1.01	−1.01	1.04	−1.02	1.03	1.06
145	6.4	60	Serum albumin = ALB	H0YA55	245	−1.59	−1.14	−1.16	1.40	1.37	−1.02
146	6.8	60	Serum albumin = ALB	P02768	116	−1.00	1.09	−1.08	1.09	−1.07	−1.17
147	7.0	60	Early endosome antigen 1 = EEA1	Q15075	28	−1.16	1.02	−1.07	1.18	1.08	−1.10
148	7.4	60	Keratin, type I cytoskeletal 9 = KRT9	P35527	182	−1.44	1.02	−1.05	1.48	1.37	−1.08
149	7.1	60	Transcriptional repressor protein YY1 fragment = YY1	H0YJV7	42	−1.14	−1.06	1.04	1.08	1.19	1.11
154	5.3	59	Actin, cytoplasmic 1 = ACTB	P60709	391	−1.43	1.10	−1.03	1.58	1.38	−1.14
165	4.2	55	Isoform 3 of Integrin alpha-IIb = ITGA2B	P08514-3	268	−1.09	1.05	1.02	1.15	1.11	−1.03
167	8.4	55	Pyruvate kinase = PKM	H3BTN5	197	1.01	−1.01	1.09	−1.02	1.07	1.10
168	8.6	55	Myeloperoxidase = MPO	J3QSF7	107	−1.33	1.03	1.05	1.37	1.40	1.02
169	8.8	55	Pyruvate kinase isozymes M1/M2 = PKM	P14618	371	−1.07	1.07	1.21	1.15	1.29	1.13
170	9.4	55	Isoform H7 of Myeloperoxidase = MPO	P05164-3	79	1.14	1.48	−1.15	1.31	−1.30	−1.70
180	5.1	54	Actin, cytoplasmic 1 = ACTB	P60709	353	−1.14	1.17	−1.12	1.33	1.01	−1.32
184	5.3	53	Actin, cytoplasmic 1 = ACTB	P60709	394	−1.32	−1.05	−1.09	1.25	1.21	−1.04
185	5.5	53	Actin, cytoplasmic 1 = ACTB	P60709	365	1.28	−1.29	−1.03	−1.65	−1.32	1.26
186	5.7	53	Actin, cytoplasmic 1 = ACTB	P60709	333	1.12	−1.21	−1.36	−1.36	−1.52	−1.12
191	6.9	52	Fibrinogen beta = FGB	P02675	107	1.18	1.12	−1.46	−1.05	−1.72	−1.64
195	7.2	51	Fibrinogen beta = FGB	P02675	163	1.07	1.01	1.01	−1.06	−1.05	1.00
196	7.0	51	Fibrinogen beta = FGB	P02675	170	1.06	1.02	−1.19	−1.04	−1.26	−1.21
197	7.5	51	Fibrinogen beta = FGB	P02675	157	−1.14	−1.05	−1.10	1.09	1.04	−1.05
199	7.3	50	Keratin, type I cytoskeletal 10 = KRT10	P13645	108	−1.11	1.05	−1.00	1.16	1.10	−1.05
200	7.8	50	Fibrinogen beta = FGB	P02675	329	−1.05	−1.12	−1.16	−1.07	−1.10	−1.03
201	7.6	50	Fibrinogen beta = FGB	D6REL8	64	−1.03	−1.05	−1.14	−1.02	−1.11	−1.09
202	8.1	50	Fibrinogen beta = FGB	P02675	232	−1.05	−1.01	−1.06	1.04	−1.01	−1.05
204	4.3	50	Nucleosome assembly protein 1-like 1 = NAP1L1	F8VRJ2	79	1.21	1.12	1.01	−1.08	−1.20	−1.11
205	8.5	50	Fibrinogen beta = FGB	P02675	336	−1.11	1.00	1.01	1.11	1.12	1.01
207	8.0	50	Cyclin-dependent kinase 4 = CDK4	F8W1L8	41	−1.02	−1.03	−1.24	−1.01	−1.22	−1.21
208	8.7	50	Isoform 2 of Fibrinogen alpha = FGA	P02671-2	101	−1.13	−1.18	−1.04	−1.05	1.08	1.13
213	7.7	49	Fibrinogen beta = FGB	P02675	156	−1.01	−1.03	−1.11	−1.01	−1.10	−1.08
214	8.1	49	Fibrinogen beta = FGB	P02675	212	−1.02	−1.06	−1.09	−1.04	−1.07	−1.03
219	4.9	48	ATP synthase subunit beta = ATP5B	H0YH81	409	−1.07	−1.03	−1.13	1.04	−1.06	−1.10
222	4.7	47	ATP synthase subunit beta = ATP5B	H0YH81	302	−1.06	1.03	−1.31	1.09	−1.24	−1.35
224	8.2	47	Isoform 2 of UTP–glucose-1-phosphate uridylyltransferase = UGP2	Q16851-2	85	−1.07	1.08	−1.24	1.16	−1.16	−1.35
226	6.7	47	Alpha-enolase = ENO1	P06733	167	1.11	1.03	1.01	−1.08	−1.10	−1.02
232	5.8	45	Actin, cytoplasmic 1 = ACTB	P60709	281	−1.12	−1.21	−1.07	−1.07	1.05	1.13
233	7.1	45	Alpha-enolase = ENO1	P06733	128	−1.12	−1.03	1.11	1.09	1.25	1.15
234	7.2	45	Alpha-enolase = ENO1	P06733	363	−1.07	−1.04	1.10	1.04	1.18	1.14
235	7.4	45	Enolase = ENO3	E5RGZ4	85	−1.23	−1.07	−1.07	1.14	1.15	1.00
236	9.1	45	Vasodilator-stimulated phosphoprotein = VASP	P50552	187	−1.11	1.09	1.16	1.21	1.29	1.06
238	5.5	45	Actin, cytoplasmic 1 = ACTB	P60709	294	1.01	−1.14	−1.06	−1.16	−1.08	1.08
240	7.6	45	Alpha-enolase = ENO1	P06733	347	−1.14	−1.05	−1.06	1.09	1.08	−1.01
241	8.8	45	Transcription factor 4 = TCF4	H3BME8	306	−1.73	−1.19	1.03	1.45	1.79	1.23
242	9.3	45	Isoform 2 of Septin-7 = SEPT7	Q16181-2	118	−1.40	1.05	1.03	1.46	1.44	−1.01
243	8.7	44	Isoform Delta of Lactotransferrin = LTF	P02788-2	206	−1.04	1.01	1.18	1.05	1.23	1.18
244	5.1	44	Actin, cytoplasmic 1 = ACTB	P60709	390	−1.29	−1.11	−1.23	1.16	1.05	−1.10
246	8.2	44	Acyl-coenzyme A synthetase mitoch = ACSM2B	H3BQ84	39	−1.17	−1.07	1.28	1.10	1.50	1.36
247	8.4	44	Alpha-enolase = ENO1	P06733	423	−1.15	1.01	1.18	1.16	1.35	1.16
254	7.3	42	Actin, cytoplasmic 1 = ACTB	P60709	318	−1.10	1.20	1.13	1.31	1.24	−1.06
255	7.5	42	Actin, cytoplasmic 2 = ACTG1	P63261	55	−1.36	1.06	−1.03	1.44	1.32	−1.09
256	7.6	42	Alpha-enolase = ENO1	P06733	166	−1.19	1.15	−1.03	1.37	1.16	−1.18
257	8.0	42	60 kDa heat shock protein, mitoch = HSPD1	E7EXB4	51	−1.30	1.08	1.01	1.40	1.32	−1.06
260	7.9	41	Elongation factor Tu, mitoch = TUFM	P49411	338	−1.41	1.20	−1.00	1.70	1.41	−1.20
261	6.6	41	POTE ankyrin domain family member F	A5A3E0	121	−1.47	1.22	−1.10	1.79	1.33	−1.34
262	7.7	41	Elongation factor Tu, mitoch = TUFM	P49411	195	−1.52	1.16	−1.18	1.76	1.29	−1.37
264	4.9	41	Actin, cytoplasmic 1 = ACTB	P60709	336	−1.05	−1.01	−1.07	1.03	−1.02	−1.06
267	6.4	41	Actin, cytoplasmic 2 = ACTG1	F5H0N0	106	−1.19	1.06	−1.26	1.25	−1.06	−1.33
268	6.7	41	Actin, cytoplasmic 1 = ACTB	P60709	298	−1.12	1.18	1.02	1.32	1.14	−1.16
269	6.9	41	Actin, cytoplasmic 2 = ACTG1	P63261	170	−1.16	1.03	1.06	1.19	1.22	1.03
270	5.0	41	Actin, cytoplasmic 1 = ACTB	P60709	383	−1.14	−1.03	−1.18	1.10	−1.04	−1.14
271	8.6	41	Isoform 2 of Fibrinogen alpha = FGA	P02671-2	72	−1.15	1.10	−1.02	1.27	1.13	−1.12
272	8.7	41	Isoform H14 of Myeloperoxidase = MPO	P05164-2	83	−1.30	−1.16	−1.13	1.12	1.15	1.02
273	8.8	41	Isoform 2 of Fibrinogen alpha = FGA	P02671-2	54	−1.18	−1.07	−1.03	1.10	1.15	1.04
275	5.2	40	Actin, cytoplasmic 1 = ACTB	P60709	418	−1.26	−1.21	−1.15	1.04	1.10	1.05
279	7.3	39	UPF0515 protein = C19orf66	K7EML3	34	−1.17	1.03	1.12	1.20	1.30	1.08
283	5.9	39	Actin, cytoplasmic 2 = ACTG1	P63261	170	−1.45	−1.01	−1.03	1.44	1.41	−1.02
296	8.1	38	Actin, cytoplasmic 2, N-terminal = ACTG1	K7EM38	36	−1.20	−1.08	−1.06	1.11	1.13	1.02
300	8.4	38	Phosphoglycerate kinase 1 = PGK1	P00558	250	−1.07	−1.19	−1.07	−1.12	1.00	1.12
303	4.5	38	Actin, cytoplasmic 1 = ACTB	P60709	91	1.17	1.23	−1.01	1.04	−1.19	−1.25
304	8.7	38	Vimentin = VIM	B0YJC4	479	−1.00	−1.15	−1.20	−1.15	−1.20	−1.04
305	9.1	38	Phosphoglycerate kinase 1 = PGK1	P00558	194	−1.07	1.06	−1.10	1.13	−1.03	−1.16
307	4.4	37	Vimentin = VIM	B0YJC4	698	1.14	1.13	1.19	−1.01	1.04	1.05
309	7.0	37	Leukocyte elastase inhibitor = SERPINB1	P30740	388	−1.07	−1.03	−1.04	1.04	1.03	−1.02
310	7.1	37	Actin, cytoplasmic 1 = ACTB	P60709	41	−1.19	1.13	1.01	1.34	1.20	−1.12
312	8.7	37	Vimentin = VIM	B0YJC4	632	1.03	−1.07	1.00	−1.10	−1.03	1.07
320	5.1	36	Actin, cytoplasmic 1 = ACTB	P60709	307	1.16	−1.37	−1.07	−1.59	−1.25	1.28
321	5.3	36	Actin, cytoplasmic 1 = ACTB	P60709	247	−1.07	−1.42	−1.02	−1.32	1.05	1.39
324	4.7	36	Isoform 2 of Tropomyosin beta = TPM2	P07951-2	258	1.15	−1.10	1.10	−1.27	−1.04	1.22
329	7.2	35	DnaJ homolog subfamily B member 11 = DNAJB11	H7C2Y5	41	−1.25	1.03	−1.06	1.29	1.17	−1.10
330	7.3	35	Cyclin-dependent kinase 4 = CDK4	F8W1L8	39	−1.16	−1.11	1.07	1.05	1.24	1.18
333	7.6	35	Cyclin-dependent kinase 4 = CDK4	F8W1L8	38	−1.19	1.05	−1.03	1.25	1.16	−1.08
335	5.6	35	Actin, cytoplasmic 1 = ACTB	P60709	225	−1.14	−1.28	−1.15	−1.12	−1.02	1.11
337	5.8	34	Actin, cytoplasmic 1 = ACTB	P60709	456	−1.20	−1.12	−1.05	1.07	1.15	1.07
339	6.3	34	Tubulin beta chain	Q5JP53	121	−1.28	−1.44	−1.19	−1.12	1.07	1.20
343	6.7	34	Actin, cytoplasmic 1 = ACTB	P60709	97	−1.17	1.14	1.09	1.34	1.27	−1.05
344	3.6	33	Stromal interaction molecule 2 = STIM2	Q9P246	33	1.43	1.06	−1.02	−1.35	−1.46	−1.07
346	9.0	33	Glyceraldehyde-3-phosphate dehydrogenase = GAPDH	E7EUT4	164	1.28	−1.09	−1.05	−1.39	−1.34	1.04
348	7.1	33	Phosphoglycerate kinase = PGK1	B7Z7A9	54	−1.23	1.08	1.19	1.32	1.45	1.10
353	8.7	32	Fructose-bisphosphate aldolase A = ALDOA	H3BMQ8	146	1.13	−1.16	−1.07	−1.31	−1.20	1.09
355	5.2	32	Talin 1 = TLN1	Q5TCU6	187	−1.13	1.00	1.27	1.14	1.44	1.26
357	8.5	32	Annexin = ANXA2	H0YN42	268	1.28	−1.05	−1.24	−1.34	−1.58	−1.18
358	4.6	31	Tropomyosin 1 (Alpha) isoform 7 = TPM1	D9YZV8	515	1.20	1.13	1.18	−1.06	−1.02	1.04
364	5.0	31	ATP synthase subunit beta = ATP5B	H0YH81	165	−1.40	−1.02	1.04	1.38	1.46	1.06
367	8.5	31	Glyceraldehyde-3-phosphate dehydrogenase = GAPDH	E7EUT4	82	1.33	−1.10	1.09	−1.46	−1.22	1.19
372	8.7	31	Heterogeneous nuclear ribonucleoproteins A2/B1 = HNRNPA2B1	P22626	226	1.02	−1.09	1.14	−1.11	1.11	1.23
373	7.3	30	Annexin A1 = ANXA1	P04083	200	−1.18	−1.13	1.27	1.04	1.49	1.44
377	8.7	30	Isoform 2 of Fibrinogen alpha = FGA	P02671-2	79	1.50	1.07	1.42	−1.40	−1.05	1.33
379	9.3	30	Fructose-bisphosphate aldolase = ALDOA	H3BQN4	176	−1.40	−1.26	−1.15	1.12	1.22	1.10
382	4.7	30	Actin, cytoplasmic 1 = ACTB	B4E335	468	1.08	−1.10	1.05	−1.19	−1.03	1.16
384	4.0	29	LVV-hemorphin-7 = HBB	F8W6P5	47	−1.09	−1.20	−1.03	−1.10	1.07	1.17
385	5.7	29	Vimentin = VIM	F5H288	171	−1.22	1.10	−1.28	1.34	−1.05	−1.40
389	6.1	28	Annexin A3 = ANXA3	P12429	549	−1.20	−1.22	1.10	−1.01	1.33	1.34
390	8.5	28	Actin-related protein 2/3 complex subunit 2 = ARPC2	O15144	64	1.09	1.01	−1.12	−1.07	−1.22	−1.14
394	6.4	27	Actin, cytoplasmic 1 = ACTB	P60709	97	−1.32	−1.22	−1.19	1.08	1.10	1.02
395	6.9	27	Keratin, type II cytoskeletal 2 epidermal = KRT2	P35908	381	−1.46	1.16	1.09	1.68	1.59	−1.06
397	4.0	27	Tropomyosin alpha-4 = TPM4	P67936	101	1.07	−1.05	−1.08	−1.12	−1.15	−1.02
400	6.7	26	Thrombospondin-1 = THBS1	P07996	769	−1.14	1.36	1.18	1.56	1.35	−1.16
403	7.2	26	Purine nucleoside phosphorylase = PNP	P00491	48	−1.01	1.04	1.07	1.05	1.08	1.03
404	7.5	26	Actin, cytoplasmic 1 = ACTB	B4DW52	133	1.23	1.16	−1.09	−1.05	−1.34	−1.27
406	8.1	26	Uncharacterized protein FLJ46347	Q6ZRH9	36	−1.07	−1.12	−1.12	−1.04	−1.05	−1.00
411	8.0	26	Unconventional myosin-IXa = MYO9A	H3BMM1	46	1.09	−1.02	−1.16	−1.11	−1.26	−1.14
421	7.4	25	Thrombospondin-1 = THBS1	P07996	368	−1.01	1.05	1.04	1.06	1.05	−1.01
423	6.9	25	Keratin, type I cytoskeletal 9 = KRT9	P35527	48	1.06	1.14	1.13	1.08	1.07	−1.01
424	4.7	25	Tropomyosin alpha-4 = TPM4	P67936	926	1.73	1.03	1.05	−1.68	−1.65	1.01
425	8.6	25	Peptidyl-prolyl cis-trans isomerase A = PPIA	P62937	110	−1.36	−1.22	−1.04	1.12	1.30	1.17
426	7.6	24	Purine nucleoside phosphorylase = PNP	P00491	154	1.42	−1.01	−1.02	−1.43	−1.44	−1.01
430	6.5	24	Thrombospondin-1 = THBS1	P07996	860	−1.10	1.01	1.25	1.11	1.37	1.24
431	6.7	24	Haloacid dehalogenase-like hydrolase domain-containing protein 2 = HDHD2	K7ER15	102	−1.20	1.19	1.32	1.43	1.58	1.11
438	8.8	23	WD repeat-containing protein 49 = WDR49	F8WBC8	46	−1.05	−1.11	1.01	−1.06	1.06	1.13
441	7.1	23	Glutathione S-transferase omega-1 = GSTO1	P78417	296	−1.12	1.14	1.10	1.28	1.24	−1.03
451	9.3	23	Glyceraldehyde-3-phosphate dehydrogenase = GAPDH	E7EUT4	71	1.02	1.17	−1.07	1.14	−1.09	−1.24
455	7.9	22	Ras suppressor protein 1 = RSU1	Q15404	89	−1.02	−1.06	−1.20	−1.03	−1.17	−1.14
461	7.0	22	Annexin A1 = ANXA1	P04083	344	1.23	−1.16	−1.02	−1.42	−1.25	1.14
462	7.3	22	Rho GTPase-activating protein 39 = ARHGAP39	Q9C0H5	31	1.19	−1.05	1.04	−1.25	−1.14	1.09
465	8.4	22	Ras suppressor protein 1 = RSU1	Q15404	204	1.17	−1.06	−1.01	−1.24	−1.17	1.06
476	4.7	21	Actin, cytoplasmic 1 = ACTB	P60709	226	1.05	−1.04	−1.07	−1.09	−1.13	−1.04
479	5.4	21	Actin, cytoplasmic 1 = ACTB	P60709	334	1.13	−1.14	−1.12	−1.29	−1.26	1.02
481	6.8	20	Peroxiredoxin-6 = PRDX6	P30041	306	−1.36	−1.09	1.03	1.25	1.40	1.12
486	7.0	20	Serum albumin = ALB	H0YA55	165	−1.45	1.11	1.06	1.61	1.54	−1.05
489	7.6	20	Keratin, type I cytoskeletal 10 = KRT10	P13645	120	−1.24	−1.07	−1.00	1.16	1.24	1.07
491	8.6	20	Thrombospondin-1 = THBS1	P07996	138	1.20	1.60	1.12	1.33	−1.07	−1.43
494	5.0	20	Actin, cytoplasmic 2, N-terminally processed = ACTG1	I3L3I4	177	1.17	1.00	−1.05	−1.16	−1.22	−1.05
495	5.1	20	Apolipoprotein A-I = APOA1	P02647	224	1.03	1.03	−1.04	1.00	−1.07	−1.08
498	6.2	20	Actin, cytoplasmic 1 = ACTB	P60709	140	−1.03	−1.47	−1.21	−1.43	−1.18	1.21
501	6.8	20	Peroxiredoxin-6 = PRDX6	P30041	237	−1.08	1.20	−1.15	1.30	−1.06	−1.38
502	7.1	20	Keratin, type II cytoskeletal 1 = KRT1	P04264	153	−1.05	1.13	−1.10	1.19	−1.05	−1.25
503	7.7	20	Myeloblastin = PRTN3	P24158	54	−1.21	−1.06	−1.09	1.15	1.12	−1.03
505	8.2	20	Thrombospondin-1 = THBS1	P07996	197	1.06	1.09	1.04	1.02	−1.02	−1.05
506	8.9	20	Parathyroid hormone 2 receptor = PTHR2	H7C0B0	42	1.17	−1.43	1.07	−1.67	−1.10	1.52
507	6.6	20	Isoform 2 of Growth factor receptor-bound protein 2 = GRB2	P62993-2	80	−1.01	1.15	−1.11	1.16	−1.10	−1.27
508	7.4	20	Serum albumin = ALB	H0YA55	224	1.12	1.02	−1.03	−1.10	−1.15	−1.05
509	8.7	20	Thrombospondin-1 = THBS1	P07996	418	−1.03	−1.01	−1.04	1.02	−1.00	−1.02
511	7.3	20	Carnitine O-palmitoyl-transferase 1, liver isoform = CPT1A	P50416	32	−1.15	−1.17	−1.05	−1.02	1.09	1.11
512	5.5	20	Actin, cytoplasmic 2 = ACTG1	P63261	150	1.14	−1.11	−1.25	−1.26	−1.42	−1.12
514	4.1	20	Transcriptional repressor protein YY1 = YY1	H0YJV7	47	1.01	1.01	−1.09	−1.00	−1.10	−1.10
515	5.3	19	Apolipoprotein A-I = APOA1	P02647	352	1.06	−1.07	−1.10	−1.13	−1.17	−1.03
518	4.3	19	Vimentin = VIM	B0YJC4	203	1.13	1.14	1.22	1.01	1.08	1.07
524	9.3	19	Keratin, type I cytoskeletal 10 = KRT10	P13645	286	−1.02	1.20	−1.13	1.23	−1.10	−1.35
530	8.6	19	Keratin, type II cytoskeletal 1 = KRT1	P04264	55	1.42	1.13	1.02	−1.25	−1.39	−1.11
531	7.2	19	Proteasome subunit beta type-3 = PSMB3	P49720	55	−1.25	1.21	1.26	1.52	1.58	1.04
533	7.1	19	Keratin, type II cytoskeletal 1 = KRT1	P04264	180	1.10	1.10	1.18	−1.00	1.07	1.07
535	8.0	19	Proteasome subunit beta type-2 = PSMB2	P49721	82	1.00	1.17	−1.05	1.17	−1.05	−1.23
540	4.3	19	Vimentin = VIM	B0YJC4	182	1.19	1.16	1.20	−1.03	1.01	1.04
544	8.4	19	Superoxide dismutase = SOD2	B3KUK2	209	1.08	1.08	1.04	−1.00	−1.04	−1.03
549	6.9	19	Glutathione peroxidase 1 = GPX1	P07203	71	1.10	1.06	1.03	−1.04	−1.07	−1.03
552	3.9	19	Isoform 3 of mitoch carrier homolog 1 = MTCH1	Q9NZJ7-3	31	1.42	1.03	−1.23	−1.38	−1.75	−1.27
554	7.1	18	Isoform 3 of mitoch. carrier homolog 1 = MTCH1	Q9NZJ7-3	38	1.27	1.38	1.53	1.09	1.21	1.11
562	6.8	18	Bestrophin-3 = BEST3	A8MVM3	38	1.26	1.31	1.42	1.04	1.13	1.09
563	6.0	18	Actin, cytoplasmic 2 = ACTG2	I3L1U9	137	−1.53	−1.74	1.44	−1.14	2.20	2.50
567	6.9	18	Glutathione S-transferase P = GSTP1	P09211	286	−1.02	−1.09	1.22	−1.07	1.24	1.33
568	3.6	18	Transcriptional repressor protein YY1 = YY1	H0YJV7	39	1.47	1.16	1.13	−1.27	−1.30	−1.03
572	5.8	18	Ferritin light chain = FTL	P02792	74	−1.01	−1.06	−1.26	−1.04	−1.25	−1.20
574	4.7	17	Tubulin beta-1 = TUBB1	Q9H4B7	39	1.21	−1.04	1.18	−1.26	−1.03	1.22
582	4.0	17	Keratin, type II cytoskeletal 1 = KRT1	P04264	116	1.34	−1.14	−1.20	−1.53	−1.60	−1.05
583	4.1	17	Isoform 3 of mitoch carrier homolog 1 = MTCH1	Q9NZJ7-3	39	−1.00	1.09	−1.07	1.09	−1.07	−1.16
588	3.9	17	Alpha-actinin-1 = ACTN1	H0YJ11	222	1.44	−1.06	−1.04	−1.53	−1.50	1.02
591	7.6	17	Heat shock 70 kDa protein 1A/1B = HSPA1A	E7EP11	43	1.01	1.26	1.42	1.25	1.41	1.13
592	4.4	17	Myosin regulatory light chain 12B = MYL12B	O14950	320	1.43	1.28	1.38	−1.12	−1.04	1.08
593	4.5	17	Actin, cytoplasmic 1 = ACTB	P60709	65	1.63	1.11	1.24	−1.46	−1.32	1.11
595	8.3	17	Protein S100-A8 = S100A8	P05109	60	1.59	−1.07	1.12	−1.70	−1.43	1.19
603	4.8	16	Annexin = ANXA5	D6RBL5	111	−1.20	1.19	1.02	1.44	1.23	−1.17
605	5.6	16	ATP synthase subunit alpha = ATP5F1A	A8K092	95	−1.03	−1.24	−1.08	−1.20	−1.05	1.14
607	6.7	16	Actin-related protein 2/3 complex subunit 3 = ARPC3	O15145	50	1.06	1.21	1.09	1.14	1.02	−1.12
610	4.5	16	Actin, cytoplasmic 2 = ACTG1	P63261	103	1.32	−1.01	1.08	−1.34	−1.22	1.10
611	7.1	16	Cofilin 1 (Non-muscle), isoform CRA_a = CFL1	G3V1A4	61	1.15	1.12	1.07	−1.03	−1.07	−1.04
612	4.2	16	Myosin regulatory light polypeptide 9 = MYL9	P24844	233	1.19	1.26	1.10	1.06	−1.08	−1.15
613	4.6	16	Actin, cytoplasmic 2 = ACTG1	P63261	61	1.45	1.04	1.13	−1.39	−1.29	1.08
614	8.7	16	Rho GDP-dissociation inhibitor 2 = ARHGDIB	H0YGX7	130	1.01	1.09	1.13	1.08	1.11	1.03
620	4.3	16	Isoform 3 of mitoch carrier homolog 1 = MTCH1	Q9NZJ7-3	38	1.33	1.00	1.22	−1.33	−1.09	1.21
621	4.6	16	Actin, cytoplasmic 1 = ACTB	P60709	153	1.20	1.02	−1.04	−1.18	−1.25	−1.06
623	5.2	16	Actin, cytoplasmic 2 = ACTG1	K7EM38	130	1.30	1.02	1.15	−1.27	−1.14	1.12
627	5.5	15	Annexin A6 = ANXA6	E5RIU8	103	−1.25	1.08	−1.27	1.35	−1.02	−1.38
628	3.8	15	Myosin light polypeptide 6 = MYL6	F8VZV5	302	1.30	1.08	1.01	−1.20	−1.29	−1.07
629	4.4	15	Actin, cytoplasmic 1 = ACTB	P60709	65	1.24	−1.07	−1.30	−1.32	−1.61	−1.22
630	8.1	15	Peptidyl-prolyl cis-trans isomerase A = PPIA	P62937	199	−1.04	−1.03	1.02	1.01	1.06	1.05
632	9.0	15	Peptidyl-prolyl cis-trans isomerase A = PPIA	P62937	235	−1.01	−1.04	−1.09	−1.03	−1.08	−1.05
637	4.4	15	Heterogeneous nuclear ribonucleoprotein K = HNRNPK	P61978	42	1.38	1.10	−1.23	−1.25	−1.70	−1.36
640	5.1	15	Actin, cytoplasmic 1 = ACTB	G5E9R0	193	1.45	1.21	1.05	−1.20	−1.39	−1.16
642	4.2	15	Actin, cytoplasmic 1 = ACTB	P60709	139	1.05	1.06	1.09	1.01	1.04	1.03
644	7.7	15	Keratin, type I cytoskeletal 10 = KRT10	P13645	47	1.16	1.18	−1.01	1.01	−1.17	−1.19
646	4.0	15	Myosin light polypeptide 6 = MYL6	F8VZV5	369	1.16	1.05	1.01	−1.11	−1.15	−1.04
648	8.4	15	Bestrophin-3 = BEST3	A8MVM3	160	1.17	1.21	1.13	1.03	−1.04	−1.07
650	7.3	15	Heterogeneous nuclear ribonucleoprotein A1 = HNRNPA1	F8W646	102	−1.04	−1.15	1.36	−1.11	1.41	1.56
657	4.8	15	Actin, cytoplasmic 1 = ACTB	P60709	108	1.25	−1.01	−1.31	−1.26	−1.63	−1.29
659	4.5	14	Actin, cytoplasmic 2 = ACTG1	P63261	255	1.37	−1.06	−1.01	−1.45	−1.39	1.04
662	4.7	14	Actin, cytoplasmic 1 = ACTB	P60709	263	1.55	1.08	−1.01	−1.43	−1.56	−1.10
664	7.0	14	Actin, cytoplasmic 2 = ACTG1	K7EM38	41	1.53	1.21	−1.02	−1.26	−1.55	−1.23
665	4.5	14	Actin, cytoplasmic 1 = ACTB	P60709	233	1.56	1.05	1.12	−1.49	−1.40	1.06
666	3.8	14	Isoform 3 of mitoch carrier homolog 1 = MTCH1	Q9NZJ7-3	32	1.34	1.07	1.12	−1.25	−1.19	1.05
671	8.7	14	Bestrophin-3 = BEST3	A8MVM3	40	1.21	1.02	−1.09	−1.19	−1.32	−1.11
673	4.0	14	Isoform H14 of Myeloperoxidase = MPO	P05164-2	288	−1.16	1.07	1.09	1.24	1.26	1.02
675	4.3	13	Actin, cytoplasmic 1 = ACTB	P60709	232	−1.05	1.04	1.10	1.09	1.15	1.05
676	4.4	13	Actin, cytoplasmic 1 = ACTB	P60709	225	1.06	−1.00	−1.01	−1.07	−1.07	−1.00
677	4.6	13	Actin, cytoplasmic 1 = ACTB	P60709	244	−1.01	−1.01	−1.17	1.00	−1.15	−1.16
679	7.7	13	Actin, cytoplasmic 1 = ACTB	C9JUM1	103	1.09	−1.31	−1.32	−1.43	−1.44	−1.01
680	7.2	13	Myosin light polypeptide 6 = MYL6	F8VZV5	421	−1.11	−1.04	−1.14	1.06	−1.03	−1.09
683	5.0	13	Histone H4 = HIST1H4A	P62805	242	1.01	−1.37	−1.43	−1.39	−1.45	−1.04
685	7.6	13	Hemoglobin subunit beta = HBB	P68871	255	−1.14	−1.55	−1.56	−1.35	−1.36	−1.00
686	4.8	13	Centromere protein H = CENPH	B3KVZ3	38	−1.19	−1.01	1.01	1.18	1.21	1.02
690	7.9	13	Hemoglobin subunit beta = HBB	P68871	384	−1.15	−1.43	−1.51	−1.24	−1.31	−1.06
696	3.8	13	Histone H4 = HIST1H4A	P62805	60	−1.31	1.04	−1.11	1.36	1.18	−1.16
697	7.8	12	Bestrophin-3 = BEST3	A8MVM3	39	1.16	−1.49	−1.53	−1.73	−1.77	−1.03
698	4.6	12	Enolase = ENO3	E5RGZ4	63	−1.24	−1.15	−1.01	1.07	1.22	1.14
703	7.7	12	LVV-hemorphin-7 = HBB	F8W6P5	41	−1.13	−1.85	−1.29	−1.64	−1.14	1.44
704	8.1	12	SH3 domain-binding glutamic acid-rich-like protein 2 = SH3BGRL2	Q9UJC5	314	1.24	−1.60	−1.31	−1.99	−1.62	1.23
706	6.7	12	Protein S100-A11 = S100A11	P31949	260	−1.39	−1.12	−1.10	1.25	1.27	1.02
707	4.6	12	Isoform 3 of mitoch carrier homolog 1 = MTCH1	Q9NZJ7-3	41	1.17	−1.20	−1.12	−1.40	−1.31	1.07
711	4.1	12	Nostrin = NOSTRIN	F8WCW8	44	−1.49	−1.07	−1.01	1.38	1.47	1.07
712	5.1	12	Myotrophin = MTPN	C9JL85	146	−1.29	−1.16	−1.66	1.11	−1.29	−1.44
713	4.9	12	Ras-related protein Ral-B = RALB	F8WEQ6	37	1.12	−1.15	−1.17	−1.29	−1.31	−1.02
718	4.5	11	Ras-related protein Ral-B = RALB	F8WEQ6	155	1.20	−1.37	−1.14	−1.65	−1.37	1.20
721	3.9	11	Protein S100-A6 = S100A6	P06703	68	−1.31	1.00	−1.11	1.31	1.18	−1.11
723	8.7	11	Transcriptional repressor protein YY1 = YY1	H0YJV7	41	1.35	−1.39	−1.15	−1.87	−1.55	1.21
725	7.7	11	Histone H4 = HIST1H4A	P62805	70	1.08	−1.30	−1.29	−1.40	−1.39	1.01
732	4.7	11	Thrombospondin-1 = THBS1	P07996	115	1.61	−1.19	−1.31	−1.91	−2.11	−1.10
735	4.3	10	SH3 domain-binding glutamic acid-rich-like protein 3 = SH3BGRL3	Q9H299	349	−1.06	1.11	−1.18	1.18	−1.11	−1.31
737	4.0	10	ATP synthase subunit alpha, mitoch = ATP5A1	K7EQT2	173	−1.16	−1.11	−1.06	1.04	1.10	1.05
738	3.9	10	Keratin, type II cytoskeletal 1 = KRT1	P04264	96	1.07	−1.10	−1.09	−1.17	−1.16	1.01
740	4.9	0	Urea transporter 1 = SLC14A1	K7EJ54	39	1.33	−1.19	−1.02	−1.59	−1.36	1.17
744	4.4	0	Ras-related protein Rap-1b = RAP1B	B4DQI8	58	−1.02	1.09	−1.11	1.11	−1.09	−1.21
745	4.2	0	SH3 domain-binding glutamic acid-rich-like protein 3 = SH3BGRL3	Q9H299	171	−1.07	−1.08	−1.08	−1.02	−1.02	1.00
756	5.3	45	Actin, cytoplasmic 1 = ACTB	P60709	343	−1.32	−1.07	−1.14	1.23	1.16	−1.06
758	5.4	38	Actin, cytoplasmic 1 = ACTB	B4E335	624	−1.30	−1.25	−1.10	1.04	1.19	1.14
759	4.8	15	Actin, cytoplasmic 2 = ACTG1	K7EM38	263	1.27	1.05	−1.02	−1.20	−1.29	−1.07
761	4.8	16	Keratin, type II cytoskeletal 1 = KRT1	P04264	789	1.24	−1.03	−1.05	−1.27	−1.30	−1.02
762	4.9	16	Actin, cytoplasmic 2 = ACTG1	P63261	221	1.34	−1.08	1.05	−1.44	−1.28	1.13
763	5.7	20	Actin, cytoplasmic 1 = ACTB	P60709	416	−1.06	−1.12	−1.10	−1.06	−1.04	1.02
769	5.4	14	Actin, cytoplasmic 1 = ACTB	C9JUM1	44	1.07	−1.06	−1.65	−1.13	−1.76	−1.56
772	5.4	13	Histone H4 = HIST1H4A	P62805	193	−1.36	−1.07	−1.18	1.27	1.15	−1.11
774	3.7	16	Calmodulin = CALM1	P62158	367	−1.11	1.13	1.10	1.25	1.22	−1.02
779	4.6	28	Actin, cytoplasmic 1 = ACTB	P60709	428	1.16	−1.01	1.02	−1.16	−1.14	1.02
781	4.6	27	Actin, cytoplasmic 1 = ACTB	P60709	342	1.04	−1.11	−1.09	−1.16	−1.14	1.01
782	4.7	27	Tropomyosin alpha-4 = TPM4	P67936	665	1.48	−1.04	1.14	−1.54	−1.31	1.18
785	9.0	13	Profilin-1 = PFN1	P07737	157	1.06	−1.13	−1.02	−1.19	−1.08	1.10
787	5.3	12	Ras-related protein Ral-B = RALB	F8WEQ6	35	−1.10	−1.01	−1.37	1.09	−1.24	−1.36
796	5.3	13	Keratin, type II cytoskeletal 1 = KRT1	P04264	127	1.29	−1.07	−1.54	−1.38	−1.99	−1.43
797	5.3	13	Keratin, type II cuticular Hb3 = KRT83	P78385	165	1.08	1.08	−1.59	1.00	−1.72	−1.72
804	7.0	10	LVV-hemorphin-7 = HBB	F8W6P5	122	1.26	−1.12	−1.28	−1.41	−1.61	−1.15
808	8.1	11	Platelet basic protein = PPBP	P02775	90	1.43	−1.33	−1.20	−1.90	−1.71	1.11
809	8.2	11	Protein S100-A8 = S100A8	P05109	66	1.23	−1.13	−1.06	−1.39	−1.30	1.07
811	8.9	11	Platelet basic protein = PPBP	P02775	216	1.25	1.02	1.05	−1.23	−1.19	1.03
816	7.5	78	Actin, cytoplasmic 1 = ACTB	B4E335	454	−1.22	1.05	−1.34	1.28	−1.10	−1.41
825	8.7	72	Isoform Delta of Lactotransferrin = LTF	P02788-2	147	−1.44	−1.07	−1.04	1.34	1.38	1.02
845	4.1	68	Isoform 4 of Regulating synaptic membrane exocytosis protein 2 = RIMS2	Q9UQ26-4	37	1.24	−1.29	−1.03	−1.61	−1.29	1.25
854	8.7	67	Kaliocin-1 = LTF	E7EQB2	46	−1.09	−1.12	−1.15	−1.02	−1.06	−1.03
863	5.5	13	Keratin, type I cytoskeletal 9 = KRT9	P35527	475	−1.79	−1.38	−1.44	1.30	1.25	−1.04
866	4.9	10	Protein S100-A6 = S100A6	P06703	39	1.46	−1.16	−1.15	−1.70	−1.68	1.01
867	4.9	0	LVV-hemorphin-7 = HBB	F8W6P5	65	1.26	−1.21	−1.19	−1.52	−1.49	1.02
868	3.6	16	Calmodulin = CALM1	P62158	343	−1.01	1.06	1.03	1.08	1.05	−1.03
870	3.6	16	Calmodulin = CALM1	E7ETZ0	63	1.45	1.11	1.09	−1.30	−1.32	−1.02
871	3.7	16	Calmodulin = CALM1	E7ETZ0	195	1.33	1.11	−1.04	−1.20	−1.39	−1.15
877	3.8	55	Stromal interaction molecule 2 = STIM2	Q9P246	39	1.31	−1.08	−1.09	−1.42	−1.43	−1.00
878	6.9	10	Protein S100-A11	P31949	246	−1.13	−1.35	−1.11	−1.20	1.01	1.22
879	6.9	11	LVV-hemorphin-7 = HBB	F8W6P5	213	−1.02	−1.22	−1.27	−1.20	−1.25	−1.04
884	4.4	22	Tropomyosin 3 = TPM3	Q5VU59	421	1.48	−1.07	1.03	−1.58	−1.43	1.10
885	4.4	21	Tropomyosin 3 = TPM3	Q5VU59	62	1.20	1.06	1.04	−1.13	−1.14	−1.02
889	7.8	70	Isoform 2 of Fibrinogen alpha = FGA	P02671-2	165	−1.04	1.07	−1.29	1.12	−1.24	−1.38
890	4.9	16	Actin, alpha 1, skeletal muscle = ACTA1	Q5T8M8	124	1.24	1.02	1.02	−1.22	−1.21	1.01
891	5.0	15	Actin, cytoplasmic 2 = ACTG1	K7EM38	113	1.23	1.05	1.02	−1.16	−1.20	−1.03
892	7.6	13	Annexin A2 = ANXA2	H0YKV8	109	−1.32	−1.37	−1.11	−1.04	1.19	1.23
896	4.3	14	Actin, cytoplasmic 1 = ACTB	P60709	198	1.15	−1.14	−1.06	−1.31	−1.22	1.07
897	4.3	14	Actin, cytoplasmic 1 = ACTB	P60709	242	1.15	1.02	1.10	−1.13	−1.05	1.08
901	8.6	13	Hemoglobin subunit beta = HBB	P68871	254	1.17	−1.26	−1.22	−1.47	−1.42	1.04
902	8.6	13	Hemoglobin subunit beta = HBB	P68871	350	1.02	−1.28	−1.19	−1.30	−1.21	1.07
903	8.6	13	Hemoglobin subunit beta = HBB	P68871	353	1.18	−1.26	−1.11	−1.49	−1.31	1.14
904	8.3	13	Hemoglobin subunit beta = HBB	P68871	349	1.07	−1.29	−1.37	−1.37	−1.46	−1.07
905	8.3	13	Hemoglobin subunit beta = HBB	P68871	330	−1.09	−1.32	−1.30	−1.21	−1.19	1.02
907	5.6	38	Actin, cytoplasmic 1 7 = ACTB	P60709	203	−1.34	−1.22	−1.06	1.10	1.26	1.14
911	4.7	10	SH3 domain-binding glutamic acid-rich-like protein 3 = SH3BGRL3	Q9H299	354	1.39	−1.02	−1.23	−1.42	−1.72	−1.21
913	4.7	117	Rho guanine nucleotide exchange factor 25 = ARHGEF25	F8W7Z4	32	1.09	−1.20	−1.07	−1.30	−1.16	1.12
914	5.1	85	Actin, cytoplasmic 2 = ACTG1	P63261	157	1.15	−1.08	1.02	−1.24	−1.12	1.10
KEY					Protein Score ≥ 62	7.00	1.00	1.00	10.00	6.00	4.00
						5.00	5.00	7.00	20.00	22.00	4.00

a *The PBMC protein samples from normal healthy (NH, n = 30), Chagas disease clinically asymptomatic (ChD CA, n = 30) and Chagas disease clinically symptomatic (ChD CS, n = 30) human subjects were incubated with (Asc+) or without (Asc-) ascorbate and resolved by 2D-GE approach. Gels were labeled with BODIPY FL N- (2-aminoethyl) maleimide, images were analyzed with SameSpots software, and normalized spot volumes were used for comparison*.

b *Proteins spots with ≥ |1.5| fold change in S-nitrosylation level (p < 0.05) in Chagas disease ChD subjects were subjected to MALDI-TOF MS/MS analysis and those identified with high confidence (score >62) are highlighted in orange color. Protein spots with low Mascot score were confirmed by LC MS/MS*.

c *The DSNO modification levels were quantified as Asc^−^/ Asc+ for each protein spot*.

d *First, ratiometric calculation from BODIPY-fluorescence units in Asc+ aliquots (normal vs. experimental) was conducted for quantifying the differential abundance of protein spots (Δprotein abundance = Asc+ ChD CA or ChD CS / Asc+ NH) was performed. The ratio of ratios, i.e., RoR = [Asc– experimental / Asc– NH] / [Asc+ experimental / Asc+ NH] was then calculated to obtain the change in S-NO levels normalized for protein abundance. As S-NO modification inhibits the Cys-BODIPY fluorescence; a negative (dark/light green) and a positive (light/dark red) RoR value would indicate an increase and decrease in S-NO levels, respectively, in experimental (vs. control) subjects*.

### Biotin Switch Assay for Verification of SNO-Modified Proteins in ChD

**Figure 4:**
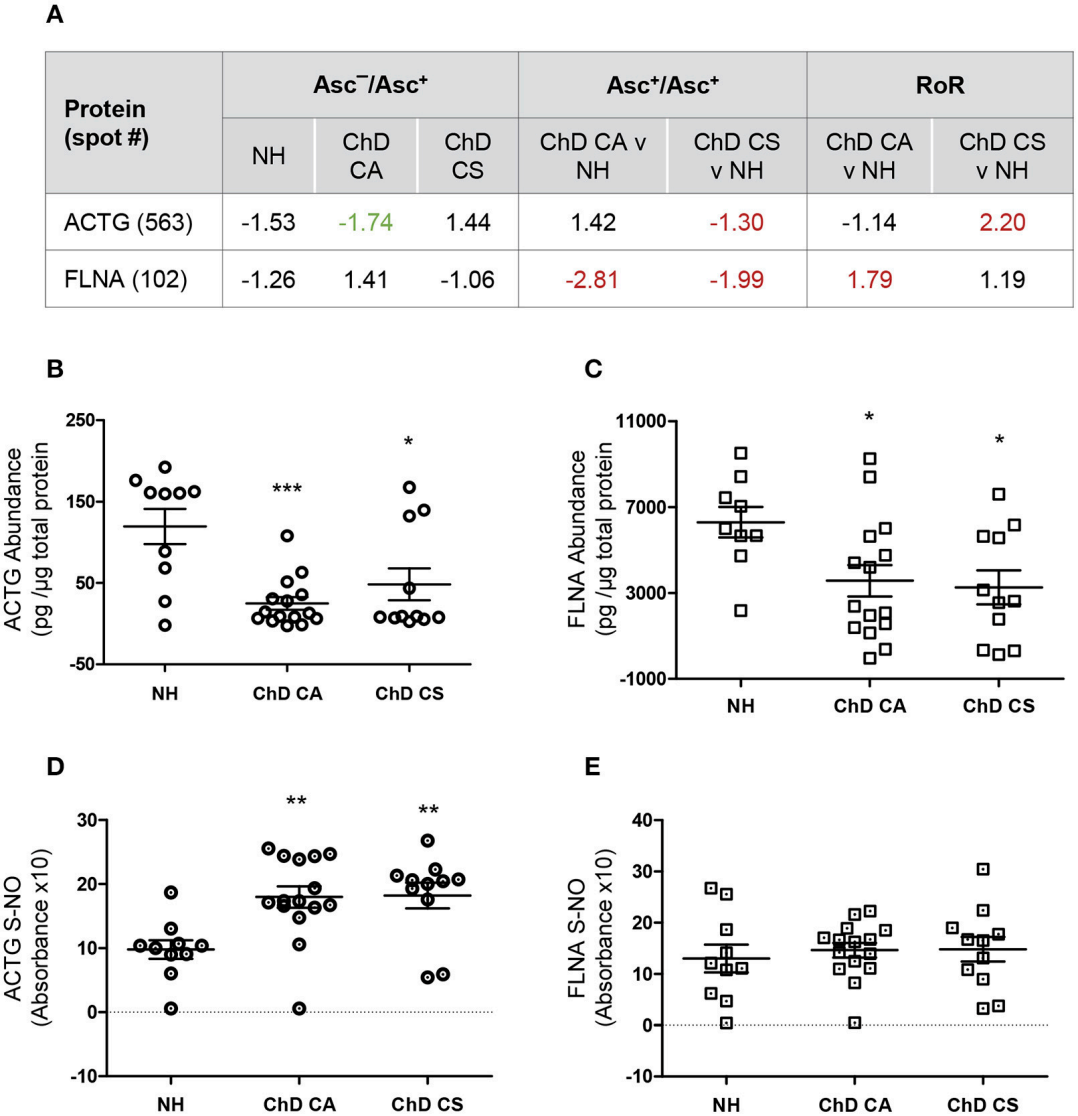
Biotin switch assay for verification of abundance and SNO levels of ACTG and FLNA in Chagas disease. **(A)** SNO proteome dataset for ACTG and FLNA are presented. **(B–E)** PBMC protein lysates (2 μg) from normal healthy (NH) controls and seropositive, Chagas Disease clinically asymptomatic (ChD CA) and Chagas disease clinically symptomatic (ChD CS) patients (*n* = 10–14 per group) were evaluated for abundance **(B,C)** and SNO **(D,E)** levels of ACTG **(B,D)** and FLNA **(C,E)** by using an ELISA-based method. For the analysis of SNO-modified proteins, PBMC lysates were subjected to biotin switch procedure prior to a modified ELISA protocol as described in section Materials and Methods. ANOVA_Tukey_ test was performed to evaluate the significance (*p < 0.05, **p < 0.01, and ***p < 0.001).

We utilized a new set of PBMC from NH, CA, and CS cohorts (*n* = 10–14 per group), and employed ELISA and biotin-switch assays to verify the changes in abundance and SNO levels of two proteins in Chagas disease. We chose filamin A and actin gamma for these studies because spot#102 and spot#563 for these proteins were found to have most changes at RoR level in ChD CA and ChD CS subjects, respectively (Figures 3C,D, 4A).

We first examined the changes in abundance of ACTG and FLNA in ChD patients and NH controls by an ELISA. In ChD CA patients (vs. NH controls), we observed 79% (*p* < 0.001) and 49.6% (*p* < 0.05) decline in the ACTG and FLNA protein levels, respectively (Figures 4A,B). In ChD CS patients, ACTG and FLNA protein levels were decreased by 59.6 and 48%, respectively (vs. NH controls, *p* < 0.05, Figures 4A,B). The finding of a decline in FLNA in ChD CA and ChD CS patients and of ACTG in ChD CS patients by ELISA was in alignment with the finding of a decline in the abundance of these proteins in the proteomic study (Asc^+ChD^/Asc^+NH^, Figure 4A). The finding of an overall decline in ACTG level in ChD CA patients by ELISA did not corroborate the proteomic finding (compare Figure 4B and Figure 4A).

For the detection of SNO modification levels of proteins in PBMC lysates, the reduced thiol groups were blocked, and then SNO-modified proteins were reduced to make these available for binding with biotin, and detection by avidin-conjugated horseradish peroxidase method. These data showed >80% changes in SNO levels of ACTG in PBMC from ChD CA and ChD CS patients (vs. NH subjects, Figure 4D). No significant changes in the SNO levels of FLNA were detected between PBMC of the three groups (Figure 4E).

The differences in the observations between ELISA/Biotin Switch Assay and BD-fluor/2D-GE based proteomic study can at least partially be explanined by the fact that 2D-GE/proteomic approach identifies the full length protein as well as derived peptides as individual spots that may change in concentration depending on their stability and/or degradation, while ELISA did not discriminate between the full length and smaller peptides of these proteins; the level of detection will depend on the epitopes that the coated antibody recognizes. Secondly, Biotin switch assay simply detects the SNO-modified proportion of a protein but does not take into consideration the changes in its abundance, while RoR approach normalizes SNO level against the protein concentration in experimental sample and then also derives the values in comparison to control data. At present no other reliable and easy to use assays exist that can detect the SNO modification level per unit protein. Despite the discrepancies between the two approaches, our results in Figure 4 suggest that use of the coating antibodies against specific ACTG and FLNA peptides that are SNO-modified will offer a more reliable diagnostic approach in distinguishing the disease state in Chagas patients.

### MARS Predictive Modeling of SNO-Modified Proteins in Chagas Disease

For this analysis, we log2 transformed the protein spot intensities to ensure that biologically relevant proportional changes are captured between the different groups (Feng et al., 2013). The log2 transformed protein spot intensities on each of the Asc^−^ and Asc+ gels of PBMC lysates from NH (*n* = 30), ChD CA (*n* = 25), and ChD CS (*n* = 28) subjects were used to calculate the Asc^−^/Asc+ ratios for each spot, and the protein spots that exhibited significant changes in the Asc^−^/Asc+ ratio (indicates relative proportion of the SNO-modified protein in the spot) at *p* ≤ 0.05_*t*−test/Welch/BH_ between any of the two groups were subjected to MARS analysis to develop the classification model.

**Figure 5:**
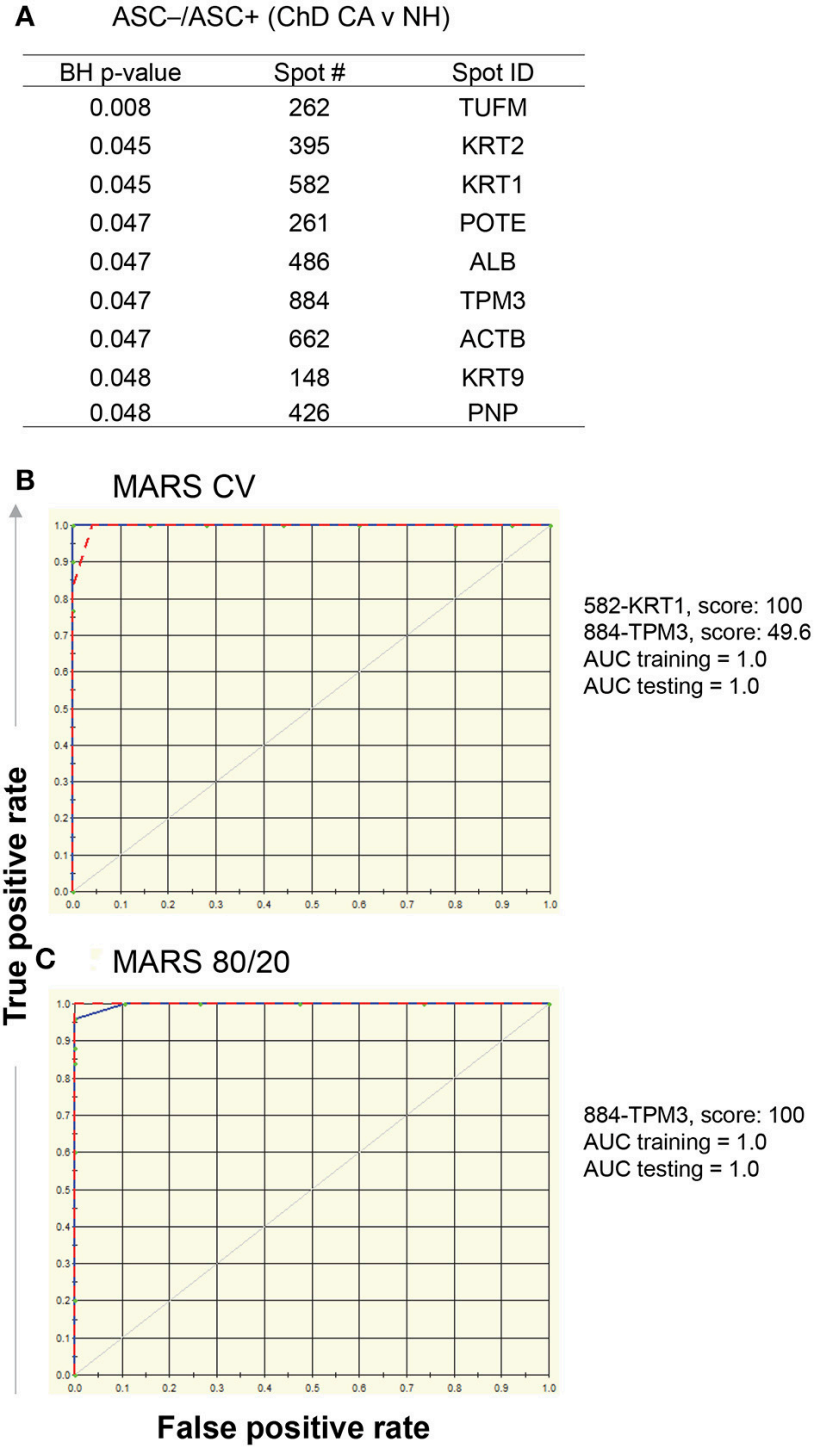
MARS modeling of SNO-modified proteins for classification of clinically asymptomatic ChD patients. For this, log2 transformed protein spot intensities on each of the Asc+ and Asc^−^ gels of PBMC lysates from ChD clinically asymptomatic (ChD CA, *n* = 25) and normal healthy (NH, *n* = 30) controls were used to calculate Asc^−^/Asc+ ratio (indicates relative proportion of the protein spot that is SNO modified). **(A)** List of protein spots showing significant changes in Asc^−^/Asc+ ratio in ChD CA patients vs. NH controls at *p*-value of <0.05_*t*−test/Welch/BH_. The 9 molecules listed in **(A)** were used as input for MARS analysis. We employed **(B)** 10-fold cross-validation (CV) and **(C)** 80% testing / 20% training approaches to assess the fit of the model for training and testing dataset. The ROC curves show the prediction success of the CV **(B)** and 80/20 **(C)** models is high for both training (blue curve) and testing (red curve) data.

Comparing Asc^−^/Asc+ values of ChD CA vs. NH groups, we identified nine protein spots that exhibited differential SNO modification level (*p* ≤ 0.05_*t*−test/Welch/BH_, Figure 5A). These nine spots were used as input to MARS modeling with 10-fold CV and 80/20 approaches. The CV MARS model ranked the nine spots with high to low priority and allocated predictive values to top ranked molecules (spot#582-KRT1 and spot#884-TPM3). The CV training (AUC/ROC = 1.0) and testing [(AUC/ROC = 1.0) models exhibited high confidence in correctly identifying the ChD CA patients (vs. NH controls] with 100% prediction success and no mistakes (Figure 5B). The 80/20 approach built the predictive model based on top ranked molecule (spot#884-TPM3). The training of this model by using datasets from randomly selected 80% of the samples from NH and ChD CA groups (AUC/ROC = 1.0) and testing of this model by using datasets from remaining 20% of the samples from the same groups (AUC/ROC = 1.0) also yielded high confidence in correctly identifying the NH and ChD CA subjects with 100% prediction success (Figure 5B).

**Figure 6:**
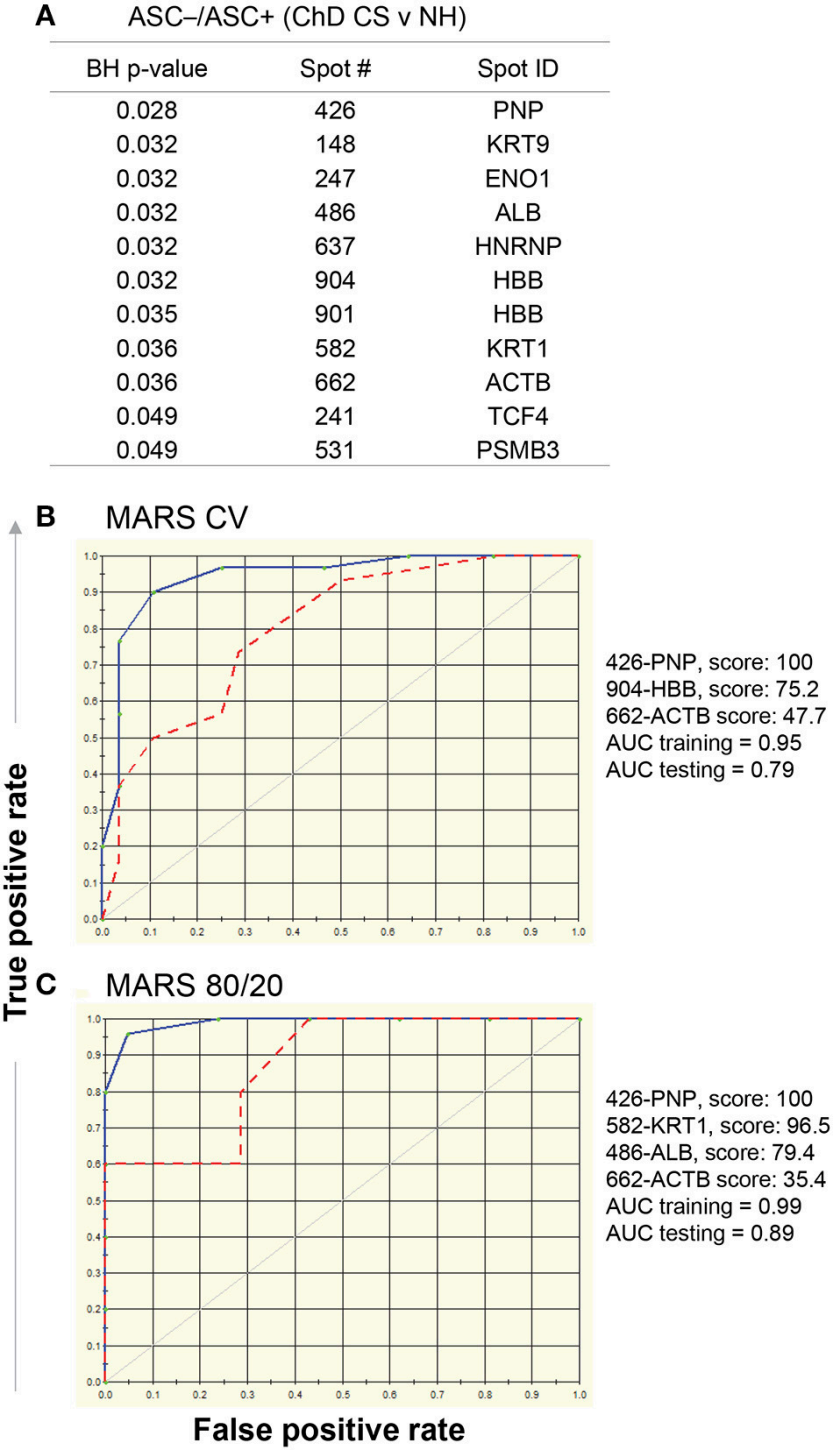
MARS modeling of SNO-modified proteins for classification of clinically symptomatic ChD patients. As in Figure 4, log2 transformed protein spot intensities on each of the Asc+ and Asc^−^ gels of PBMC lysates from ChD clinically symptomatic (ChD CS, *n* = 28) patients and normal healthy (NH, *n* = 30) subjects were used to calculate Asc^−^/Asc+ ratios for each protein spot **(A)** List of protein spots showing significant changes in Asc^−^/Asc+ ratio in ChD CS patients (vs. NH controls at *p*-value of <0.05_*t*−test/Welch/BH_. The 11 molecules listed in **(A)** were used as input for MARS analysis. **(B)** The ROC curves show the prediction success of the 10-fold cross validation model is high for the training data but not for the testing data. **(C)** The ROC curves for 80/20 model show good prediction success for the training and testing data. Blue curve: training data, red curve: testing data.

Comparing Asc^−^/Asc+ values of ChD CS vs. NH groups, we identified 11 protein spots that exhibited significant differences in SNO modification level (*p* < 0.05_*t*−test/Welch/BH_, Figure 6A). These eleven spots were used as input to MARS modeling with 10-fold CV and 80/20 approaches. The CV MARS model ranked the eleven spots with high to low priority and allocated predictive values to top ranked molecules (spot#426-PNP, spot#904-HBB, and spot#662-ACTB). The CV training model correctly identified 90% and 92.86% of the NH and ChD CS subjects, respectively, with overall prediction success of 91.38% (AUC/ROC = 0.95, Figure 6B). However, the CV model performed poorly in testing phase, and correctly identified only 73.3 and 64.3% of the NH and ChD CS subjects, respectively (AUC/ROC = 0.79, Figure 6B). The 80/20 approach chose four top-ranked molecules (spot#426-PNP, spot#582-KRT1, spot#486-ALB, and spot#662-ACTB), and this model performed better than the CV model in predicting the ChD CS vs. NH subjects. The training of this model by using datasets from 80% of the NH and ChD CS subjects yielded high confidence (AUC/ROC = 0.99, Figure 6C) with an overall prediction success of 82.61% and correctly identified 100% of ChD CS subjects. The testing of this model with datasets from 20% of the NH and ChD CS subjects also yielded good confidence (AUC/ROC = 0.89, Figure 6C) with an overall prediction success of 83.33%.

Together, the results presented in Figures 5, 6 suggest that a) SNO modification of KRT1 and TPM3 peptides will have high predictive value in diagnosing the ChD CA status of infected patients; and b) SNO modification of PNP HBB, KRT1, ACTB, and ALB peptides will have high predictive value in identifying the ChD CS status of infected patients. Further, the predictive modeling worked more robustly in identifying the ChD CA subjects than in recognizing the ChD CS status. Furthermore, the protein spots predicted by this analysis were in accordance with significative RoR levels showed in Figures 3C,D (vs. Figure 5 and Figure 6, respectively). The MARS did not yield high degree of success in distinguishing the ChD CA vs. ChD CS groups (data not shown).

### Ingenuity Pathway Analysis of Chagas Disease Associated SNO Proteome Signature

**Figure 7:**
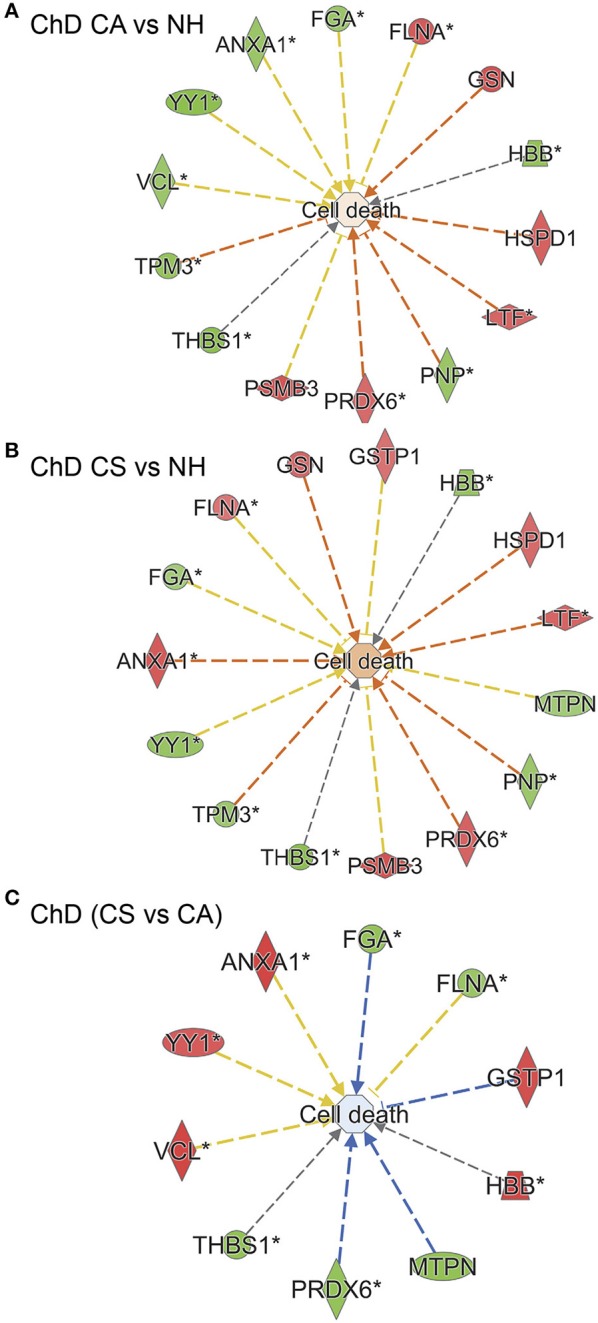
SNO profile of cell death network in Chagas disease (ChD). PBMC SNO proteome of ChD clinically asymptomatic (ChD CA, *n* = 25) and ChD clinically symptomatic (ChD CS, *n* = 28) patients and of normal healthy controls (NH, *n* = 30) was developed as described in section Materials and Methods. The RoR values for protein spots that were differentially SNO-modified (normalized to change in protein abundance) at *p*-value of <0.05 were uploaded in the ingenuity Pathway Analysis (IPA) software, and proteins that changed in RoR at fold change: |≥ 1.2| were included in biological modeling by IPA. Shown are molecules that are predicted to be associated with cell death and that were significantly changed in RoR values in **(A)** ChD CA patients vs. NH controls, **(B)** ChD CS patients vs. NH controls, and **(C)** ChD CS vs. ChD CA patients. In the networks, the intensity of red and green colors show the extent of decrease and increase in protein SNO modification, respectively. Brownish orange node/lines and blue node/lines show predicted activation and inhibition, respectively, of a pathway. Gray and yellow lines are used when the putative effect is not completely understood.

We employed Ingenuity Pathway Analysis software to determine molecular and biological functions, as well as the important pathways and networks involved in Chagas disease development. Input to IPA were the RoR values (*p* ≤ 0.05_*t*−test/Welch/BH_, Table 1) and we established a cut-off of |fold change|: ≥ 1.2 to ensure sufficient molecules are available to build the disease-related networks. IPA analysis showed that several molecules that are predicted to be associated with cell death were significantly changed in their RoR values in ChD CA (vs. NH) subjects (14 molecules, *p* = 1.89E-02, Figure 7A). Likewise cell death associated 15 molecules exhibited significant changes in RoR values in ChD CS (vs. NH) subjects (*p* = 9.6E-03, Figure 7B). The cell death network was predicted to be more responsive to SNO modification level of proteins (ANXA1, FGA, FLNA, GSTP1, HBB, MTPN, PRDX6, THBS1, VCL, YY1) in clinically symptomatic patients as compared to the clinically asymptomatic ChD subjects (*p* = 8.71E-03, Figure 7C).

**Figure 8:**
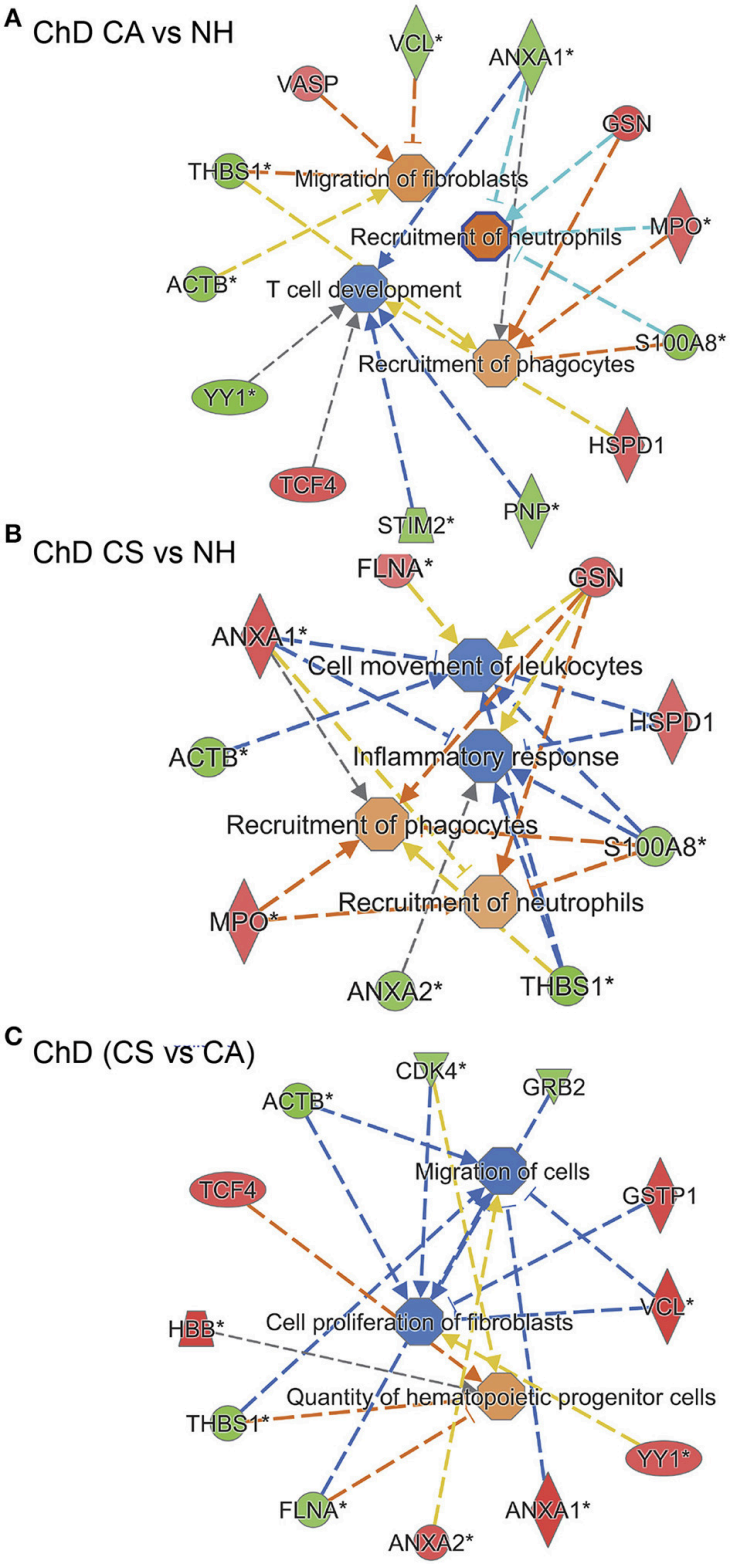
SNO profile of inflammation and immune responses network in Chagas disease. As in Figure 6, PBMC SNO proteome of ChD clinically asymptomatic (ChD CA, *n* = 25) and ChD clinically symptomatic (ChD CS, *n* = 28) patients and of normal healthy controls (NH, *n* = 30) was developed as described in section Materials and Methods. The RoR values for protein spots that were differentially SNO-modified (normalized to change in protein abundance) at *p*-value of <0.05 were uploaded in the ingenuity Pathway Analysis (IPA) software, and proteins that changed in RoR at fold change: |≥ 1.2| were included in biological modeling by IPA. Shown are molecules that are predicted to be associated with proliferation and recruitment of immune cells and fibroblasts and that were significantly changed in RoR values in **(A)** ChD CA patients vs. NH controls, **(B)** ChD CS patients vs. NH controls, and **(C)** ChD CS vs. ChD CA patients. In the networks, the intensity of red and green colors show the extent of decrease and increase in protein SNO modification, respectively. Brownish orange node/lines and blue node/lines show predicted activation and inhibition, respectively, of a pathway. Gray and yellow lines are used when the putative effect is not completely understood.

IPA analysis of RoR proteome datasets also predicted a putative differential SNO profile of proteins involved in recruitment of immune cells, i.e., leukocytes, neutrophils, and phagocytes, and overall development of inflammatory response in ChD CA patients (13 molecules, *p*: 1.84E-02 to 3.26E-04, z score range: 0.9–1.954, Figure 8A) and ChD CS patients (9 molecules, *p*: 2.37 E-03 to 4.52E-03, z score range: 0.8–1.387, Figure 8B) when compared to the NH controls. The extent of SNO modification of proteins predicted to contribute to the proliferation of HEMATOPOIETIC cells (ACTB, CDK4, GRB2, GSTP1, VCL, YY1, ANXA1, ANXA2, FLNA, THBS1, HBB, and TCF4) was more pronounced in ChD CS (vs. ChD CA) patients (*p*: 5.12E-03, z score: 1.0, Figure 8C). However, a –ve z score values indicated that S-nitrosyation of proteins in ChD CS (vs. ChD CA) patients may be used to control the acute phase like signaling (FGA, GRB2, RALB, VCL, z score: −2.449, *p* = 5.28E-07), cell proliferation of fibroblasts and migration of cells (z scores: −1.471 and −1.659, respectively, *p*: 3.37E-02). Together the results presented in Figures 6, 7 suggest that changes in SNO modification levels might serve as an important mechanism in regulating cell death, cell proliferation, and the development of inflammatory and fibrotic responses with progression of heart disease in Chagas disease patients.

## Discussion

In this discovery proteomic study, we have focused on identifying the S-nitrosylation profile of PBMC from seropositive ChD patients with or without clinical disease in comparison with seronegative healthy subjects (*n* = 25–30 per group). We ran 166 2D gels to resolve the protein samples and utilized BODIPY® FL *N*- (2-aminoethyl) maleimide labeling to detect changes in SNO modification in PBMC samples. Of the 635 protein spots that were detected by 2D-GE, 312 protein spots exhibited significant differences in abundance and/or SNO-modification levels (*p* ≤ 0.05) between any of the two groups, and 249 of these protein spots were successfully identified with high confidence (Table 1). Further, 30 and 28 protein spots were differentially SNO-modified (RoR fold change |≥ 1.5|, *p* < 0.05) in ChD CA and ChD CS patients, respectively (Table 1 and Figure 3). Eight of these molecules (732-THBS1, 697-BEST3, 808-PPBP, 866-S100A6, 424-TPM4, 704-SH3BGRL2, 582-KRT1, and 723-YY1) were significantly increased in SNO modification levels (indicated by negative RoR values) in ChD CA as well as ChD CS patients. We postulate that increase in SNO modification of these molecules in a ChD CA patient would serve as a warning for the development of Chagas cardiomyopathy, and absence of SNO of these eight molecules would indicate the seropositive subject is not at risk of heart disease development. This hypothesis will need to be tested in future studies.

We noted increased SNO modification (–ve RoR value) of three proteins (THBS1, S100A6, abd SH3BGRL2) in ChD patients in this study, as well in congestive heart failure (CHF) patients of idiopathic etiology in a previous study (Koo et al., 2016). Conversely, BEST3, PPBP, and YY1 exhibited opposing pattern, with a decreased s-nitrosylation in CHF patients (Koo et al., 2016) and an increase in SNO modification in ChD patients (Table 1). These findings indicate the common and distinct features of s-nitrosylation in the development of HF of diverse etiologies. Also interesting is the presence of PSMB3 as part of SNO-proteome signature of ChD subjects but not found in our previous CHF study. This is suggestive of a post-translational modification induced by the presence of the parasite. Further studies should be performed in the future in order to validate this hypothesis.

We performed MARS analysis of the datasets to understand the diagnostic potential of the SNO proteome datasets in identifying the disease status in Chagas patients (Figures 5, 6). MARS creates models based on piecewise linear regressions. It searches through all predictors to find those most useful for predicting outcomes and then creates an optimal model by a series of regression splines called basis functions (Sun et al., 2007; Benedet et al., 2018). For this, MARS uses a two-stage process; the first half of the process involves creating an overly large model by adding basis functions that represent either single variable transformations or multivariate interaction terms. In the second stage, MARS successively deletes basis functions, starting with the lowest contributor in order of least contribution to the model until the optimum model is reached. The end result is a classification model based on single variables and interaction terms that will optimally determine class identity (Sun et al., 2007; Benedet et al., 2018). The MARS analysis with two approaches to avoid overfitting of the datasets showed high-to-moderate confidence in predictive value of differential SNO modification of select proteins in identifying ChD CA (KRT1 and TPM3) and ChD CS (PNP, KRT1, ALB, HBB, ACTB) patients (Figures 4, 5). One limitation of these observations is that MARS models were built on the Asc^−^/Asc+ ratios of the protein spots, and did not consider the changes in abundance of the respective proteins. As yet, our data provide the framework for designing the multiplex diagnostic assays targeting change in abundance and SNO modification levels of the host proteins in diagnosing the exposure to parasite, disease state, and cure post-treatment. Indeed, in recent years, key s-nitrosylation targets important in cardiovascular pathophysiology of diverse etiologies were identified (reviewed in Maron et al., 2013). For example, SNO of several proteins of RyR2 and L-type Ca^+2^ channels was found to be associated with modulation of myocardial contractility, electromechanical function, and ventricular fibrillation in congestive heart failure (Burger et al., 2009), while others have shown the s-nitrosylation of mitochondrial proteins was protective against ischemia and reperfusion injury (Sun et al., 2007; Murray et al., 2011). Other investigators showed potential value of nitroyslation/denitrosylation status in identifying the clinical outcomes after septic shock in experimental models, though specific target proteins were not identified (Benedet et al., 2018). Some researchers have employed proteomic approach and found the s-nitrosylation of proteins important for synapse function, metabolism, and Alzheimer's disease pathology in the brain tissue during early stages of neurodegeneration (Seneviratne et al., 2016). We propose that the future longitudinal studies with larger patient cohorts will justly assess and confirm the potential value of the SNO-modified protein spots identified in this study in diagnosing the progression of cardiac disease in Chagas patients.

Ingenuity Pathway Analysis of the proteome datasets (RoR values, fold change |≥1.2|, *p* < 0.05) pointed to the importance of SNO modification of several molecules involved in proliferation, recruitment and migration of immune cells and fibroblasts, and in cell death pathway during the development and progression of Chagas disease (Figures 7, 8). A change in SNO modification of proteins involved in cellular disassembly and disorganization associated with disruption of filaments that is central to remodeling of the cytoskeleton and modulation of cell shape for migration was observed in PBMC of all ChD patients (Figure 8). Whether s-nitrosylation of proteins involved in immune cell proliferation, migration and cell death play a cardioprotective or cardiotoxic role in Chagas disease remains to be seen in future studies. However, we specifically discuss the proteome profile of two proteins. Our data showed the SNO as well as abundance profiles of beta and gamma isoforms of actins (ACTB, ACTG) that are the highly conserved proteins of the cytoskeleton and coexist in most cell types, that are responsible for maintaining the cell integrity, and also are mediators of internal cell motility (Chang and Goldman, 2004) were altered in PBMC of ChD patients (Table 1, Figure 8, Garg et al., 2016). Likewise, several isoforms of filamin A (FLNA), an actin binding protein that links actin filaments to membrane glycoproteins, that interacts with several molecules (e.g., integrins, transmembrane receptor complexes, and second messengers) (Nakamura et al., 2011), and that is shown to have effects on cell shape and cell migration, were also altered by s-nitrosylation in PBMC of ChD patients (Table 1). A recent study has also shown that filamin A, through its interaction with Drp1 (modulator of mitochondrial dynamics), attenuates the mitochondrial hyperfission and cardiomyocytes' senescence in an animal model of myocardial infarction (Nishimura et al., 2018). We have shown the mitochondrial dysfunction of electron transport chain, and mitochondrial production of reactive oxygen species (ROS) was exacerbated in Chagas disease (Wen and Garg, 2008, 2010; Lopez et al., 2018), and mtROS provided signal to NFκB-dependent activation of proinflammatory response in immune and non-immune cells (Ba et al., 2010; Gupta et al., 2011). These observations allow us to propose that (a) SNO modification of a select panel of proteins, specifically actins and filamins, determines the activation, migration and survival of immune cells in the circulatory system of Chagas patients, and (b) proinflammatory activation and/or senescence/death of immune cells by the ROS produced by mitochondria in the cardiac environment determines the clinical outcomes in the infected individuals with the progression of Chagas disease.

In summary, we have presented unbiased SNO proteomic analysis of PBMC of Chagas disease patients in this study. We have identified the possible pathologic mechanisms in disease progression that involve immune cell activation and cell death. MARS-modeling identified a panel of protein spots that if monitored in infected individuals, would have high degree of success in predicting risk of clinical disease development.

## Author Contributions

MZ collected and processed the human samples. MZ, JW, HS, S-JK, and NG performed experiments, analyzed data, and wrote manuscript. NB, AN, JN, VB, FI and RL recruited patients, performed physical/clinical exam, serology, and other examination, and clinically characterized patients, everyone was involved in study design, and reviewing the manuscript. NG provided financial support for carrying out the experiments. RL and NB supported the patient exam.

### Conflict of Interest Statement

The authors declare that the research was conducted in the absence of any commercial or financial relationships that could be construed as a potential conflict of interest.
